# Methionine-Homocysteine Pathway in African-American Prostate Cancer

**DOI:** 10.1093/jncics/pkz019

**Published:** 2019-04-25

**Authors:** Jie H Gohlke, Stacy M Lloyd, Sumanta Basu, Vasanta Putluri, Shaiju K Vareed, Uttam Rasaily, Danthasinghe Waduge Badrajee Piyarathna, Hunter Fuentes, Thekkelnaycke M Rajendiran, Tiffany H Dorsey, Chandrashekar R Ambati, Rajni Sonavane, Balasubramanyam Karanam, Salil Kumar Bhowmik, Rick Kittles, Stefan Ambs, Martha Pritchett Mims, Michael Ittmann, Jeffrey A Jones, Ganesh Palapattu, Nagireddy Putluri, George Michailidis, Arun Sreekumar

## Abstract

African American (AA) men have a 60% higher incidence and two times greater risk of dying of prostate cancer (PCa) than European American men, yet there is limited insight into the molecular mechanisms driving this difference. To our knowledge, metabolic alterations, a cancer-associated hallmark, have not been reported in AA PCa, despite their importance in tumor biology. Therefore, we measured 190 metabolites across ancestry-verified AA PCa/benign adjacent tissue pairs (n = 33 each) and identified alterations in the methionine-homocysteine pathway utilizing two-sided statistical tests for all comparisons. Consistent with this finding, methionine and homocysteine were elevated in plasma from AA PCa patients using case-control (AA PCa vs AA control, methionine: *P* = .0007 and homocysteine: *P* < .0001), biopsy cohorts (AA biopsy positive vs AA biopsy negative, methionine: *P* = .0002 and homocysteine: *P* < .0001), and race assignments based on either self-report (AA PCa vs European American PCa, methionine: *P* = .001, homocysteine: *P* < .0001) or West African ancestry (upper tertile vs middle tertile, homocysteine: *P* < .0001; upper tertile vs low tertile, homocysteine: *P* = .002). These findings demonstrate reprogrammed metabolism in AA PCa patients and provide a potential biological basis for PCa disparities.

Studies focusing on the biological mechanism of prostate cancer (PCa) disparities are underrepresented despite reports indicating that African American (AA) PCa patients exhibit higher tumor volume and involvement, in addition to lower 5-year biochemical recurrence survival, than their European American (EA) counterparts (1–3). To address this gap in knowledge, we examined metabolite levels in AA PCa using three distinct cohorts of deidentified clinical samples, all obtained with institutional review board approval and appropriate patient consent. These included (1) matched pairs of AA PCa (n = 33, all genetic ancestry examined) and adjacent benign tissues from a prostatectomy cohort (Supplementary Table 1, available online); (2) plasma and urine samples from age-matched AA and EA participants with extensive clinical, epidemiological, and dietary data (refer to Supplementary Methods, available online, for questionnaire) recruited into the NCI-Maryland Prostate Cancer Case-Control Study (4) (plasma: 52 AA PCa and 51 EA PCa with 25 AA and 23 EA control subjects, respectively; 66 AA and 58 EA were genetic ancestry verified, Supplementary Table 2, available online; urine: 20 AA PCa and 18 EA PCa with 19 AA and EA control subjects; 27 AA and 31 EA were ancestry verified; Supplementary Table 3, available online); and, (3) plasma obtained from a biopsy cohort of AA patients (43 biopsy negative and 80 biopsy positive; all genetic ancestry verified; Supplementary Table 4, available online). Genetic ancestry estimates were determined using Ancestry Informative Markers. Tissue samples were examined for 190 metabolites using mass spectrometry-based multiple reaction monitoring (MRM) (Figure 1A; Supplementary Tables 5 and 6, available online, show MRM transitions) using pooled samples to monitor reproducibility (coefficient of variation of pool samples are described in Supplementary Table 7, available online). Metabolic profiles were examined using a two-sided paired *t* test to determine altered metabolites, and Network Gene Set enrichment Analysis [NetGSA (5)] to identify pathways in AA PCa relative to benign adjacent tissue. Relative levels of key altered metabolites, namely methionine and homocysteine, were examined in plasma and urine samples using MRM and analyzed for statistical significance using a two-sided Wilcoxon test. Linear regression-based multivariable analysis was used on the plasma data to study associations between methionine and homocysteine levels with West African ancestry in PCa cases. Expression of homocysteine metabolizing enzymes, Betaine-Homocysteine S-Methyltransferase (*BHMT*) and cystathionine beta synthase (*CBS*), were examined using tissue microarray analysis (TMA), immunostained with commercially available *BHMT* and *CBS* antibodies. TMAs were scored for both intensity and extent of staining on a scale of 0 to 9, by a genitourinary pathologist, and was analyzed using a two-sided Wilcoxon test. In all cases, including NetGSA, Benjamini-Hochberg method was used to compute the false discovery rate (FDR). A detailed description of all methods can be found in the Supplemental section (available online).

**Figure 1.**
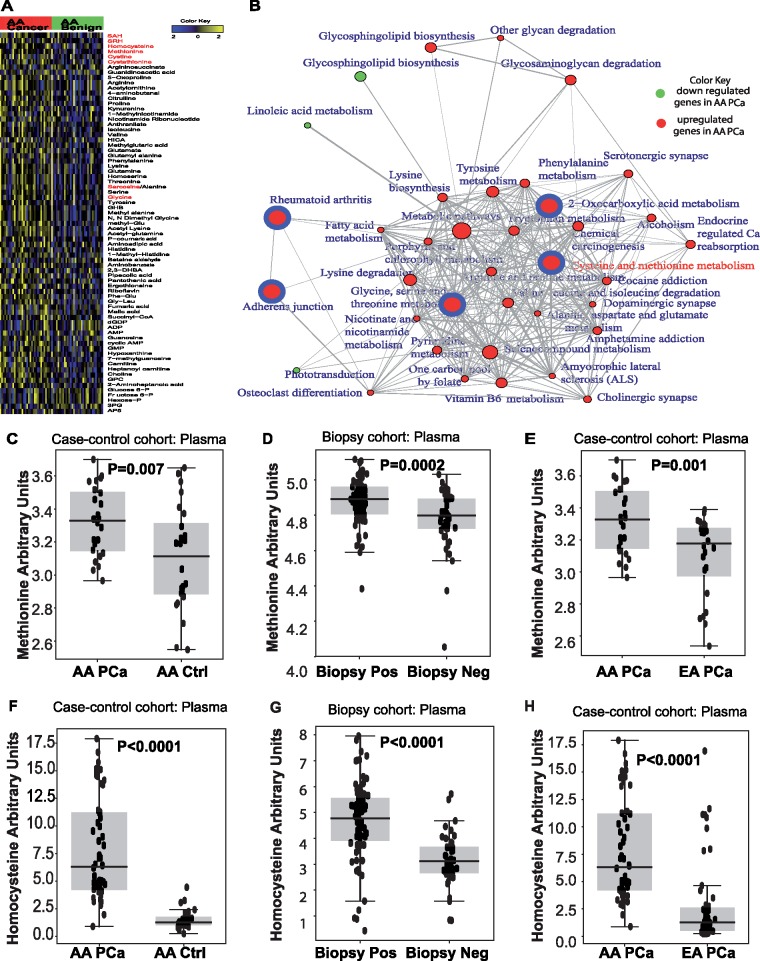
Methionine-homocysteine levels in African American (AA) prostate cancer (PCa). (**A**) Heat map showing altered metabolites in localized PCa (n = 33) and matched adjacent benign tissue in ancestry-verified AA patients. Shades of **yellow** and **blue** represent an increase or decrease of a metabolite relative to median metabolite levels (false discovery rate [FDR] = 0.3), respectively (refer to color key). Metabolites marked in **red** belong to the methionine-homocysteine pathway. (**B**) Network-based Gene Set Analysis map showing differential metabolic pathways between AA PCa and adjacent benign tissue. Pathways enriched at FDR-corrected *P* value less than .001 are shown in **solid circles**. The circumference of the circle is correlated to the pathway connectivity. **Red and green circles** indicate enriched pathways that are up- and downregulated in AA PCa, respectively. **Red circles with blue borders** indicate the most enriched pathways (*P* < 10^−5^). Association between enriched pathways are delineated by the bridges. Components of the methionine-homocysteine pathway are indicated in **red font**. Boxplots comparing the plasma methionine levels between (**C**) AA PCa and control subjects (n = 24 each), (**D**) AA biopsy-positive (n = 78) samples and biopsy-negative control subjects (n = 43), and (**E**) AA and EA PCa (n = 24 each). Boxplots comparing the plasma homocysteine levels between (**F**) AA PCa (n = 52) and control subjects (n = 25), (**G**) AA biopsy positive (n = 80) and biopsy negative (n = 43), and (**H**) AA PCa (n = 52) and EA PCa (n = 51). Two-sided *P* values for all the comparisons were calculated using Wilcoxon rank sum test.

A total of 73 of 190 metabolites examined were statistically significantly altered (*P* < .05, FDR ≤ 0.3) in AA PCa relative to adjacent benign tissue (refer Supplementary Table 8, available online for raw *P* values). This included higher levels of metabolites in the methionine-homocysteine and other amino acid pathways (Figure 1A, red font). NetGSA of all statistically significant metabolites (*P* < .05, FDR ≤ 0.3) revealed multiple amino acid pathways, with cysteine-methionine metabolism being the most enriched biochemical pathway (Figure 1B;Supplementary Table 9, available online).

In light of the above findings, we examined levels of methionine and homocysteine in plasma obtained from case-control and biopsy cohorts. Interestingly, median circulating levels of methionine and its by-product homocysteine were statistically significantly elevated in AA PCa cases vs control subjects (methionine: 3.34 [range = 2.97–3.77] vs 3.11 [range = 2.55–3.65], *P* = .007, Figure 1C; and homocysteine: 6.31 [range = 0.89–17.90] vs 1.27 [range = 0.24–4.44], *P* < .0001, Figure 1F; refer to Supplementary Figure 1, available online, for data in genetic ancestry-verified samples). Similar analyses revealed a statistically significant increase in median methionine and homocysteine levels in AA biopsy positive vs biopsy negative control subjects (methionine: 4.89 [range = 4.38–5.1] vs 4.80 [range = 4.05–5.03], *P* = .0002, Figure 1D; and homocysteine: 4.78 [range = 0.43–7.96] vs 3.10 [range = 0.83–5.72], *P* < .0001, Figure 1G; refer Supplementary Figure 2, available online, for data on biopsy cohort after 2 years of follow-up). Furthermore, levels of these metabolites in AA PCa were statistically significantly higher than EA PCa (methionine: 3.34 [range = 2.97–3.77] vs 3.16 [range = 2.54–3.39], *P* = .001, Figure 1E; and homocysteine: 6.31 [range = 0.89–17.90] vs 1.26 [range = 0.23–16.92], *P* < .0001, Figure 1H). Methionine and homocysteine levels were not statistically significantly different between EA PCa and control subjects or AA control subjects and EA control subjects (Supplementary Figure 3, available online).

To evaluate potential associations between West African ancestry and metabolite levels, we stratified the case-control cohort into tertiles based on percent West African ancestry. Homocysteine levels were statistically significantly (*P* < .0001) elevated in the upper (n = 38 self-reported AA, median = 4.57 [range = 0.54–16.67]) vs middle tertile (n = 46, 30 self-reported AA and 16 self-reported EA, median = 1.57 [range = 0.24–17.90]) and upper vs bottom tertile (n = 41 self-reported EA, median = 1.46 [range = 0.23–16.92], *P* = .002, Figure 2A). In addition, we also carried out a nonparametric Spearman Rank Correlation analysis to obtain insights into potential associations between elevated levels of methionine and homocysteine with West African ancestry in an unstratified manner. Interestingly, we observed a positive correlation between methionine levels and West African ancestry only in PCa cases (rho = 0.44, *P* = .01; Supplementary Figure 4A, available online). Homocysteine levels were also correlated with West African ancestry across all samples (rho = 0.46, *P* < .001; Supplementary Figure 4B, available online) and among PCa cases (rho = 0.45, *P* < .0001; Supplementary Figure 4C, available online).

**Figure 2.**
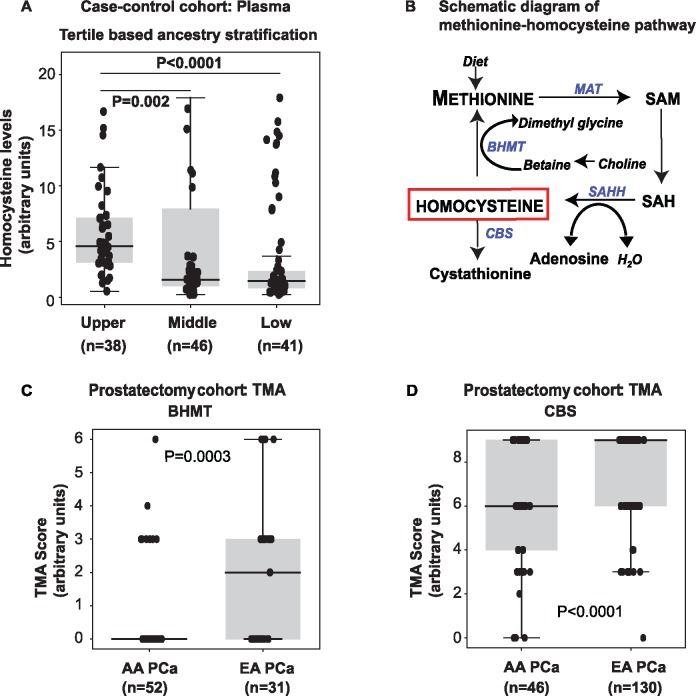
West African ancestry and plasma homocysteine, and the expression of homocysteine metabolizing enzymes, in African American (AA) prostate cancer (PCa). (**A**) Comparison of plasma homocysteine in case-control samples stratified into tertiles based on their West African ancestry. (**B**) Schematic diagram of methionine-homocysteine pathway. Homocysteine can be metabolized to cystathionine by cystathionine beta synthase (CBS) or remethylated back to methionine by Betaine-Homocysteine S-Methyltransferase (BHMT). (**C**) Tissue microarray analysis (TMA) for BHMT in AA PCa (n = 52) and EA PCa (n = 31). (**D**) Same as in C, but for CBS (AA PCa: n = 46 vs EA PCa: n = 130). Two-sided *P* values for all the panels were calculated using Wilcoxon rank sum test. MAT = Methionine adenosyltransferase; SAM = S-adenosylmethionine; SAH: S-adenosylhomocysteine; SAHH: S-adenosyl-L-homocysteine hydrolase.

Because methionine is an essential amino acid, additional analyses of dietary data collected within the case-control study were examined to determine if this could account for the observed differences in circulating levels of methionine and homocysteine between AA and EA samples. A multivariable linear regression analysis controlling for body mass index, age at diagnosis, socioeconomic status (education, income, and number of individuals in the household), and either meat doneness or fish or bacon fat consumption was done separately for PCa cases and control subjects. This analysis further confirmed that in PCa cases, self-reported AA race is independently associated with plasma methionine and homocysteine levels (Supplementary Tables 10–12, available online). This relationship was weaker among the control subjects. Other factors that showed associations with plasma methionine and homocysteine levels included body mass index, income, and meat doneness (Supplementary Tables 10–12, available online).

Methionine is converted to homocysteine, which is either remethylated back to methionine by *BHMT* or committed to the trans-sulfuration pathway by *CBS* (Figure 2B). Tissue microarray analysis was performed on tissues obtained from a prostatectomy cohort and revealed statistically and biologically significant reductions of *BHMT* (*P* = .0003; Figure 2C), and *CBS* (*P* < .0001; Figure 2D) in AA PCa relative to EA PCa, thereby validating accumulation of homocysteine in AA PCa (Supplementary Figure 5, available online, shows the cumulative frequency of TMA scores).

Elevated levels of homocysteine are consistent with earlier findings that describe increased methylation in AA PCa (6), which is reflected by higher levels of sarcosine, an N-methyl derivative of the amino acid glycine, in AA PCa compared with benign adjacent tissue (Figure 1A). Statistically significant increases in median levels of sarcosine were also found in urine samples (clinical data in Supplementary Table 3, available online) of all PCa vs control subjects (0.05 [range = 0.01–0.50] vs 0.03 [range = 0.005–0.15]). Sarcosine levels were also statistically higher in AA PCa vs AA control subjects (AA PCa: 0.06 [range = 0.01–0.5] vs AA control subjects: 0.03 [range = 0.005–0.15]) and AA PCa vs EA PCa (0.04 [range = 0.015–0.26]; Supplementary Figure 6, available online).

Notably, higher homocysteine levels are known to promote the formation and degeneration of bone matrix (7), which are integral events associated with PCa-associated bone metastasis (8). It is thus intriguing to speculate that higher levels of homocysteine found in AA patients with PCa may promote tumor cell homing to the bone, a premise that warrants further investigation.

In summary, for the first time we have shown that both self-reported and ancestry-stratified AA PCa is characterized by alterations in the methionine-homocysteine pathway, resulting in accumulation of methionine and homocysteine in tissue, plasma, and a clinically relevant biopsy patient population. In support of these findings, elevated homocysteine levels were associated with West African ancestry in men with PCa in a case-control setting. Lastly, in AA PCa, the expression of enzymes responsible for recycling and trans-sulfuration of these metabolites was statistically and biologically significant reduced. Overall, this first-in-field metabolic assessment of AA PCa provides biological insights that could contribute to PCa disparities.

## Funding

The authors acknowledge the joint participation of the Diana Helis Henry Medical Research Foundation through its direct engagement in the continuous active conduct of medical research in conjunction with the Baylor College of Medicine (BCM) and the Global Center for Mass Spectrometry Excellence supported by Agilent Technologies at BCM. This research was partially supported by the following grants: NIH U01 CA167234 (ASK, GP, and GM), NIH UO1CA179674-01 (ASK) W81XWH-12-1-0046 (MI), W81XWH-11-1-0737 (MPM),  Prostate Cancer Foundation Challenge Award (ASK, JAJ, and MI), Brockman Medical Research Foundation (SML, ASK) ACS 127430-RSG-15-105-01-CNE (NP), R01CA216426 (NP), Dan L. Duncan Cancer Center (P30 CA125123) supporting Human Tissue Acquisition and Pathology, and Metabolomics Shared Resources, CPRIT Metabolomics Core Facility (RP170005) supporting VP, CRA, NP and ASK, and Intramural Research Program of the Center for Cancer Research (ZIA BC010499 and ZIA BC 010624, SA). JHG was supported by National Institute of Health, Baylor College of Medicine Oncology scholars T32 fellowship T32CA174647. JHG is currently a Diana Helis Henry Medical Research Foundation fellow.

## Notes

Affiliations of authors: Department of Molecular and Cellular Biology and Dan L Duncan Comprehensive Cancer Center, Baylor College of Medicine, Houston, TX (JHG, SML, VP, SKV, UR, DWBP, CRA, RS, SKB, NP, AS); Verna and Marrs McLean Department of Biochemistry and Molecular Biology (AS); Adrienne Helis Melvin Medical Research Foundations, New Orleans, LA (JHG); Department of Biological Statistics and Computational Biology, Cornell University, Ithaca, NY (SB); Michael E. DeBakey Veteran Affairs Medical Center and Department of Urology, Baylor College of Medicine, Houston, TX (HF, JAJ); Department of Pathology, University of Michigan, Ann Arbor, MI (TMR); Laboratory of Human Carcinogenesis, Center for Cancer Research, National Cancer Institute, Bethesda, MD (THD, SA); Department of Biology and Cancer Research, Tuskegee University, Tuskegee, AL (BK); Division of Health Equities, City of Hope Comprehensive Cancer Center, Duarte, CA (RK); Department of Medicine-Hematology & Oncology (MPM) and Department of Pathology and Immunology (MI), Baylor College of Medicine, Houston, TX; Department of Urology and Rogel Comprehensive Cancer Center, University of Michigan, Ann Arbor, MI (GP); Department of Urology, Medical University of Vienna, Vienna, Austria (GP); Department of Statistics, University of Florida, Gainesville, FL (GM).

The authors declare no conflict of interest.

JHG, SML, SB, GM, and AS conceived and designed the study. JHG, SML, SB, VP, SKV, UR, DWBP, TMR, HF, RS, BK, THD, CRA, RK, MI, and SA developed the methodology and acquired the data. JHG, SML, SB, SA, GP, NP, GM, and AS wrote, reviewed, and/or revised the manuscript. THD, SKB, SA, CRA, JAJ, and MPM provided administrative, technical, or material support (i.e., reporting or organizing data, constructing databases). AS supervised the study.

## Supplementary Material

Supplementary Data
