# Supplementary material for: Methionine-Homocysteine Pathway in African-American Prostate Cancer
Source: JNCI Cancer Spectr. 2019 Apr 25;3(2):pkz019. doi: 10.1093/jncics/pkz019 (PMC6489686; doi:10.1093/jncics/pkz019)
Supplement: Supplementary Data [file pkz019_supp.pdf]

Supplementary Material

Methionine-homocysteine pathway in African-American prostate cancer

Jie H. Gohlke[1,2,*], Stacy M. Lloyd[1*], Sumanta Basu[3*], Vasanta Putluri[1], Shaiju K. Vareed[1], Uttam Rasaily[1] , Danthasinghe Waduge Badrajee Piyarathna[1], Hunter Fuentes[4], Thekkelnaycke M. Rajendiran[5], Tiffany H. Dorsey[6], Chandrashekar R. Ambati[1], Rajni Sonavane[1], Balasubramanyam Karanam[7], Salil Kumar Bhowmik[1], Rick Kittles[8], Stefan Ambs[6], Martha Pritchett Mims[9], Michael Ittmann[10], Jeffrey A. Jones[4], Ganesh Palapattu[11], Nagireddy Putluri[1], George Michailidis[12], Arun Sreekumar[1] #

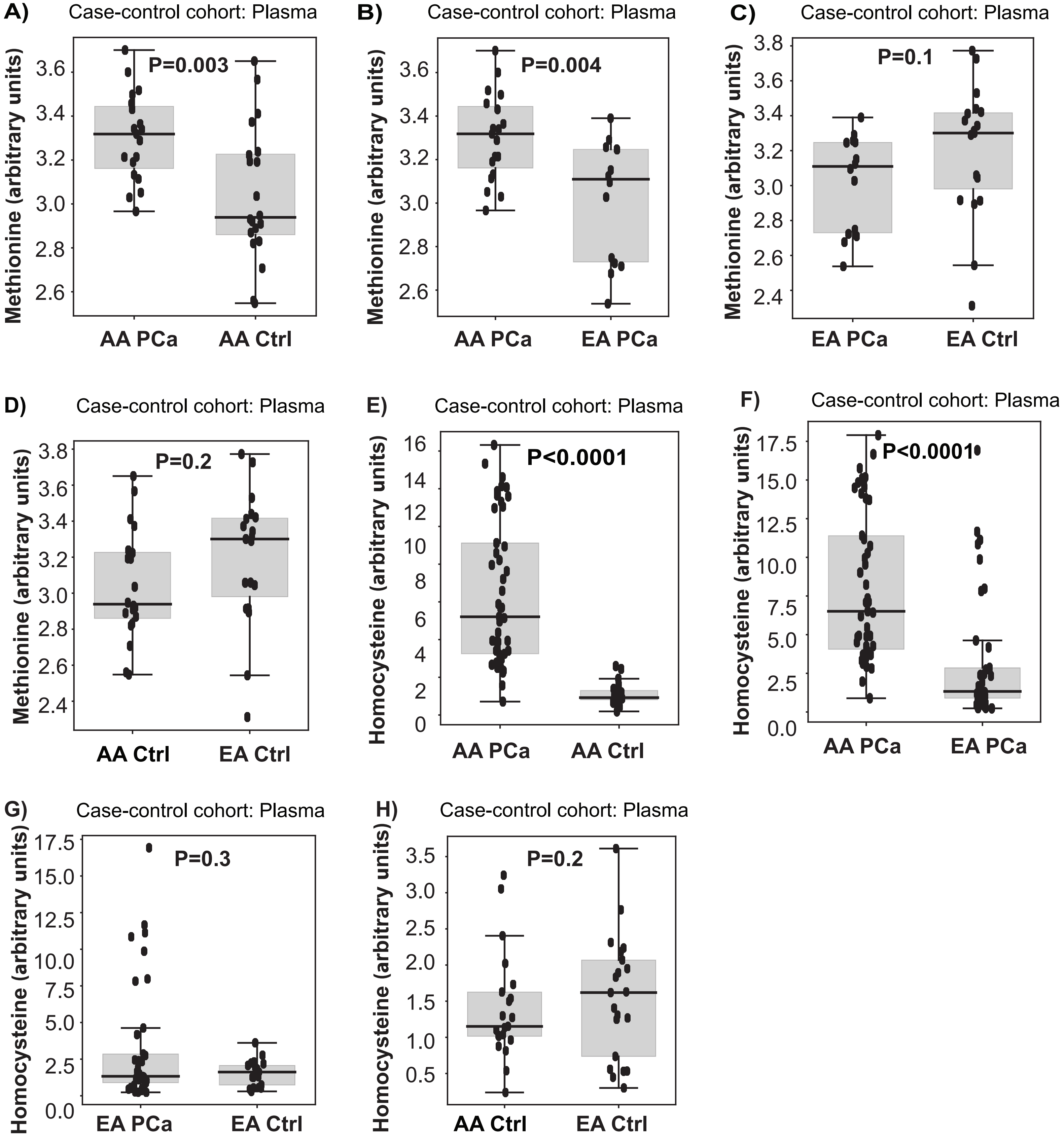

Supplementary Figure 1. Plasma methionine and homocysteine levels in genetic ancestry-verified case-control samples. Boxplots comparing the plasma methionine levels between A) AA PCa cases and AA controls (AA PCa: n = 19 and controls: n = 20), B) AA and EA PCa cases (EA PCa: n = 14), C) EA PCa cases and EA controls (n = 19), D) AA controls and EA controls. Boxplots comparing the plasma homocysteine levels between E) AA PCa cases and AA controls (AA PCa: n = 45; controls: n = 21), F) AA and EA PCa cases (EA PCa: n = 37), G) EA PCa case and EA controls (EA PCa: n = 37), H) AA controls and EA controls (EA controls: n = 21). P-values for all comparisons computed using Wilcoxon rank sum test.

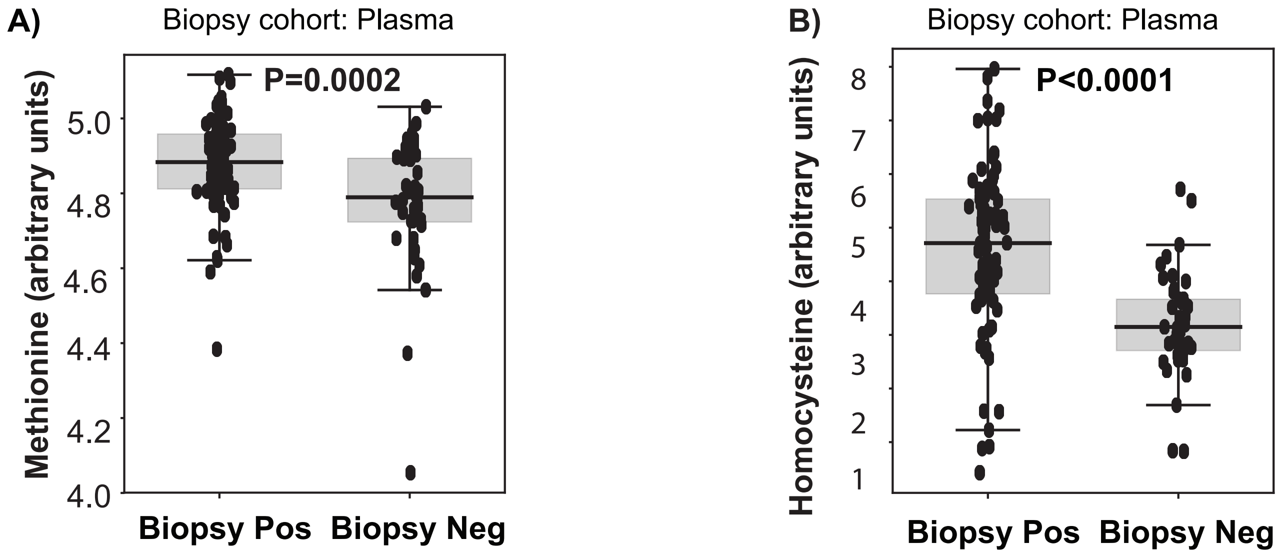

Supplementary Figure 2. **Plasma methionine and homocysteine levels in AA biopsy cohort after two years of clinical follow up.** A) methionine levels in biopsy positive PCa samples (n = 80) compared to biopsy negative controls (n = 41). B) homocysteine levels in biopsy positive (n = 82) compared to biopsy negative controls (n = 41). Importantly, these are the same samples used in Figures 1 D and G with the exception that here the biopsy negative status for two patients were revised to biopsy positive PCa after two years of clinical follow up. Rest of the biopsy negative controls remain disease free at the end of two-year follow up period. Therefore, compared to data shown in Figure 1, the methionine measurement includes two additional biopsy positive and two less biopsy negative samples. For homocysteine measurement, there are two additional biopsy positive and two less biopsy negative samples. Importantly, irrespective of the change in clinical status for the two biopsy negative patients, both methionine and homocysteine levels remained significantly elevated compared to biopsy negative controls. Significance levels for all comparisons was computed using Wilcoxon rank sum test.

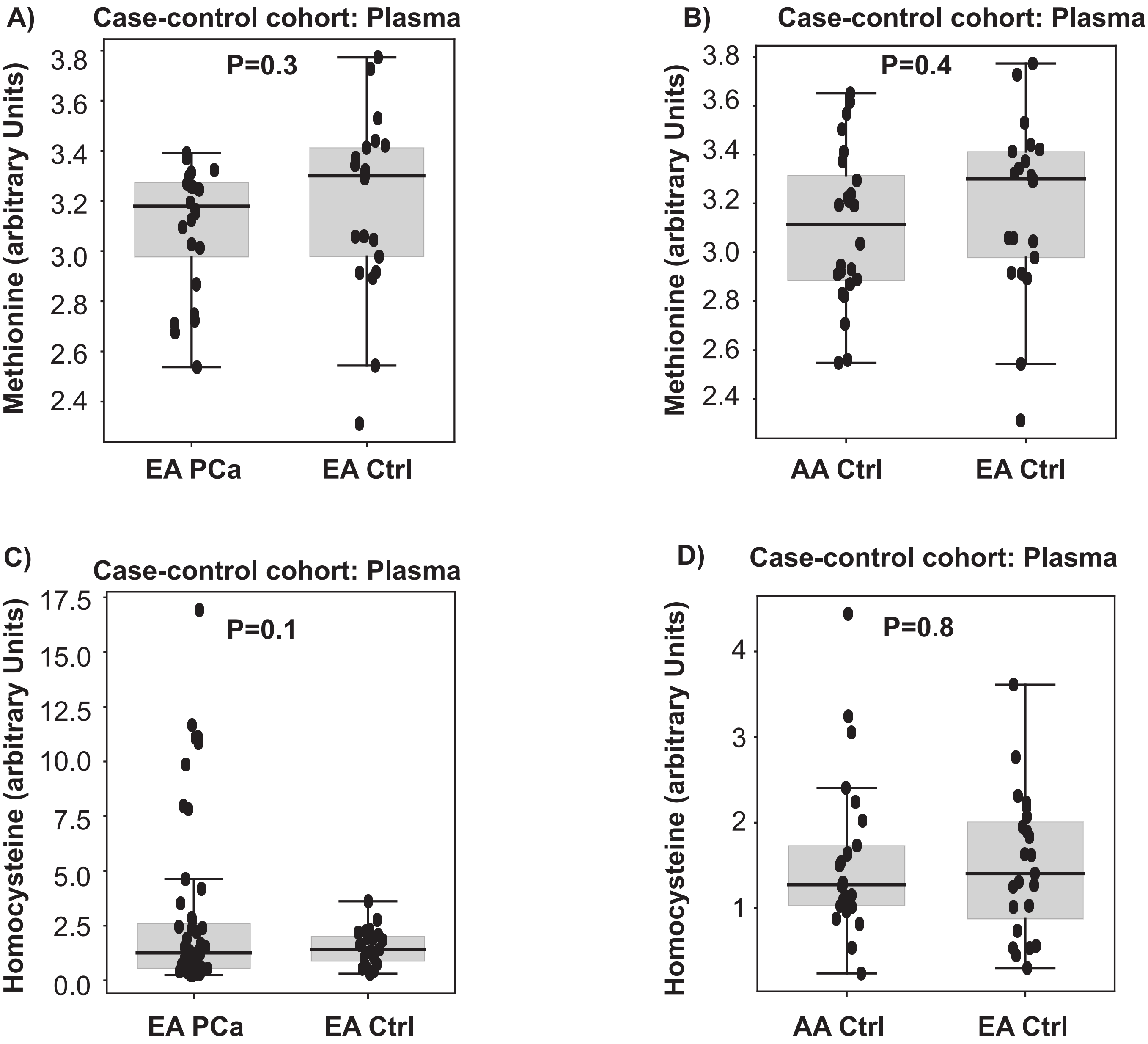

Supplementary Figure 3. **Plasma methionine and homocysteine levels in EA PCa cases vs controls and AA vs EA controls.** A) Plasma methionine levels in EA PCa cases (n = 24) compared to EA controls (n = 21). B) same as in A, but for AA controls compared to EA controls (AA controls n = 24). C) same as in A, but for homocysteine levels in EA PCa cases (n= 51) vs EA controls (n = 21). D) Same as in B, but for homocysteine levels in AA controls vs EA controls. Significance levels for all comparisons was computed using Wilcoxon rank sum test.

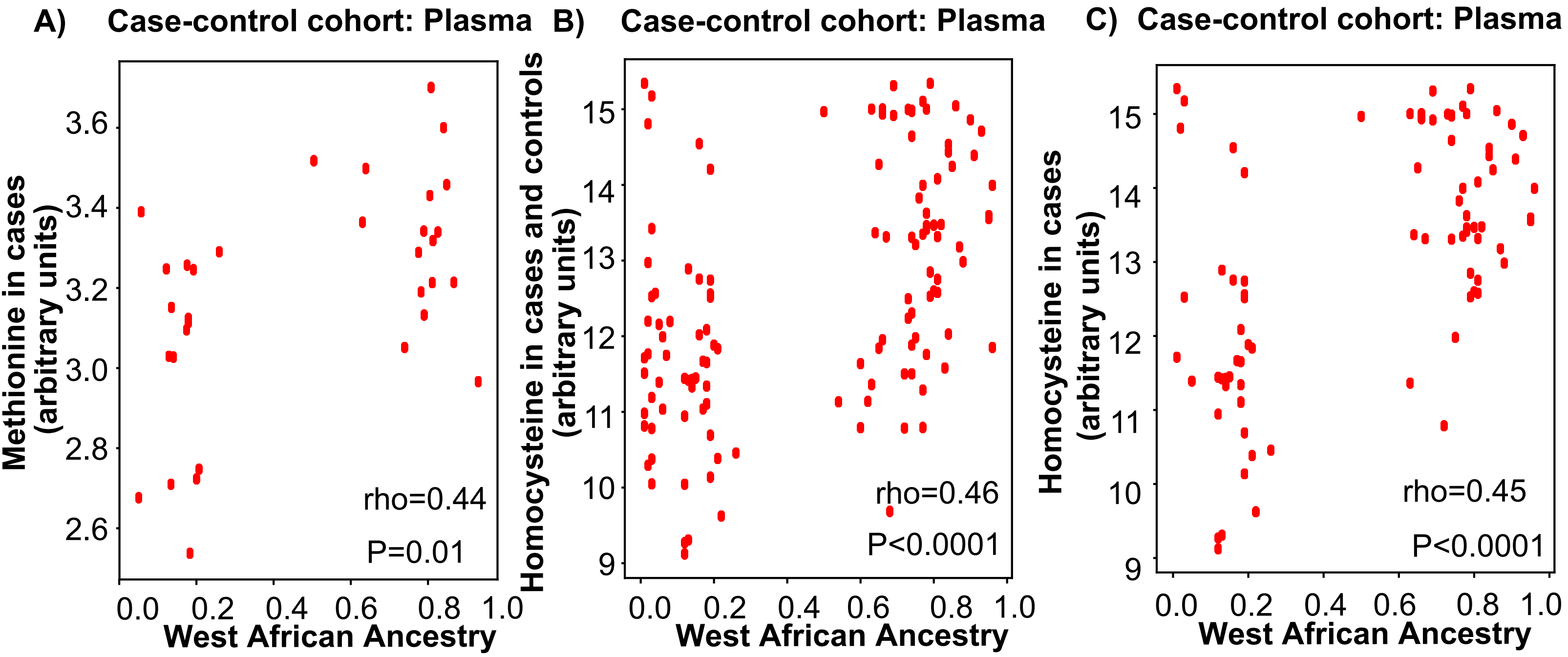

Supplementary Figure 4. Scatter Plots showing the results of Spearman Rank Correlation analysis examining the association between West African ancestry and plasma levels of metabolites within the case-control cohort. A) Scatter plot showing the association between plasma methionine levels and West African ancestry in the PCa cases, B) same as in A, but for homocysteine and West African ancestry in PCa cases and controls, C) same as in B, but in PCa cases.

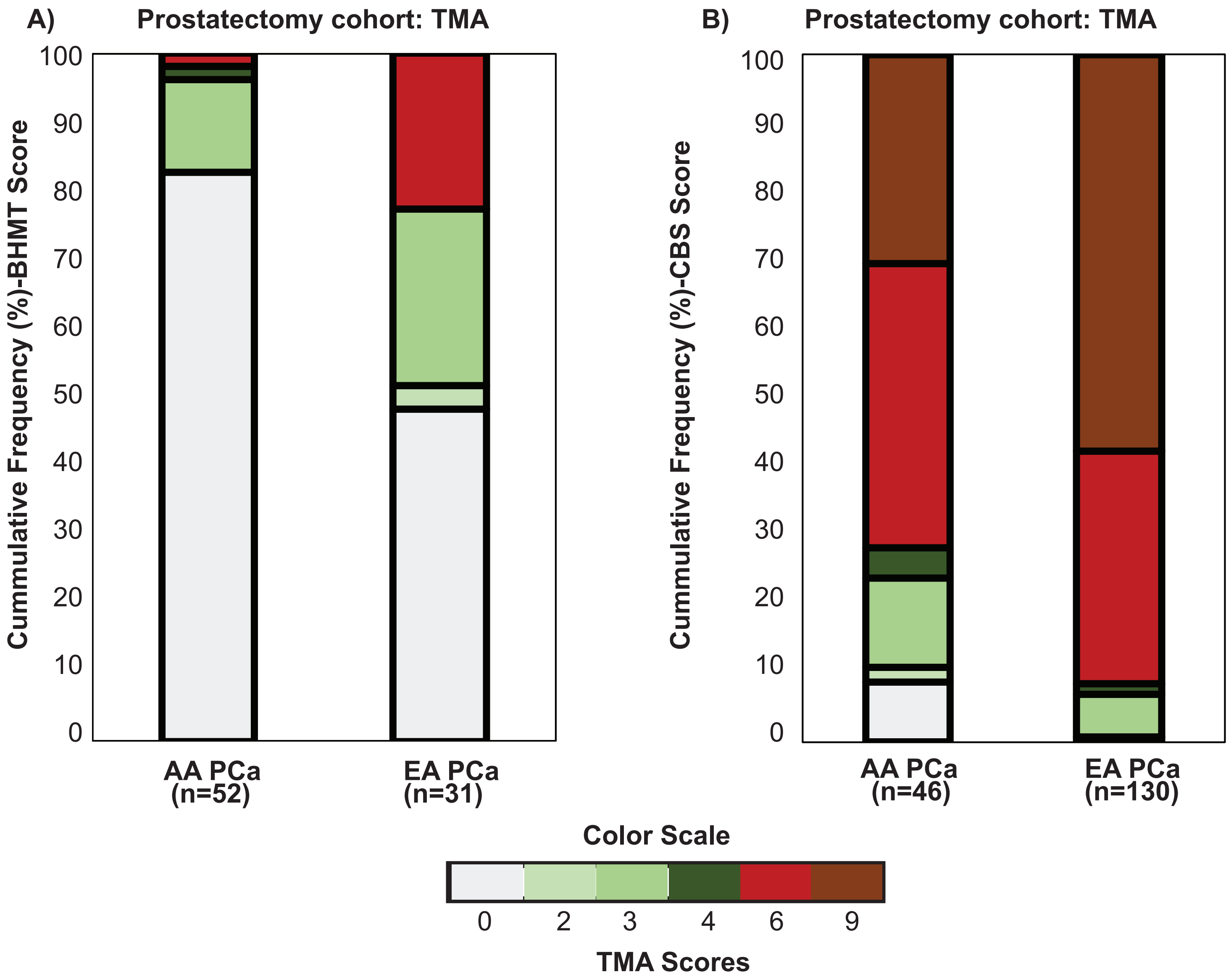

Supplementary Figure 5. Cummulative bar plots showing the frequency distribution of immunostaining scores in tissue microarrays. A) for Betaine Hydroxymethyl Transferase (BHMT), and B) for Cystathionine Beta Synthase (CBS). Refer to color scale for colors corresponding to the different scores.

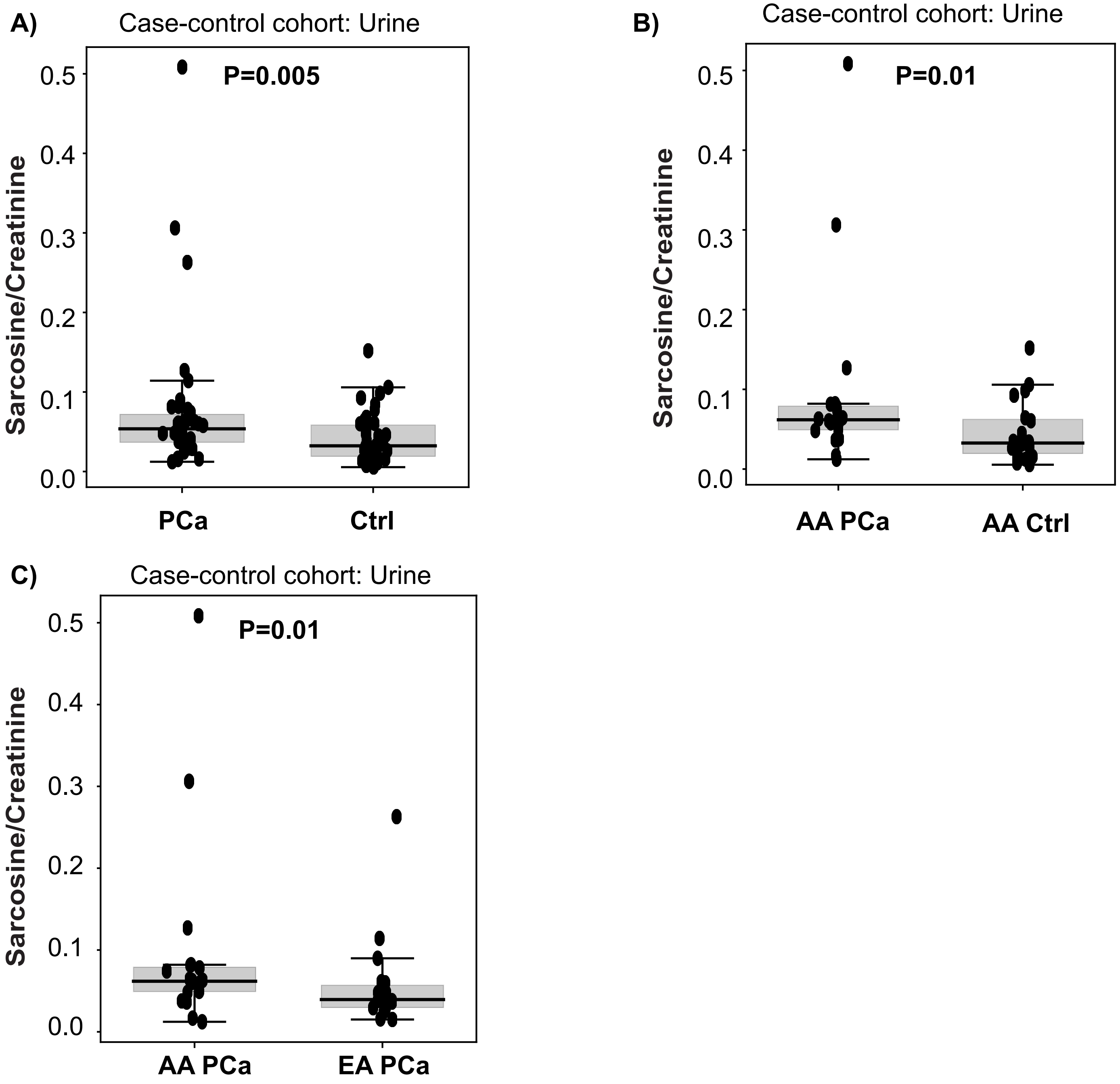

Supplementary Figure 6. **Sarcosine to Creatinine ratio in urine of self reported PCa cases and controls from the NCI case-control cohort.** A) Urine sarcosine to creatinine ratio in PCa cases (n = 38) compared to controls (n = 38). B) Urine sarcosine to creatinine ratio in AA PCa cases (n = 20) compared to AA controls (n = 19). C) Urine sarcosine to creatinine ratio in AA PCa cases (n = 20) compared to EA PCa cases (n = 17). Wilcoxon rank sum test was used to generate the P values.

Supplementary Table 1. Summary of clinical and ancestry data for prostatectomy tissue cohort (metabolomics)

Variable	African American (n=33)
Gleason Grade	Low (≤6): 8
	High (≥7): 25
Recurrence	7*
West African, ŷ	0.8 ± 0.1#
European, ŷ	0.2 ± 0.1
Native American, ŷ	0.0 ± 0.0
Genetic Ancestry Tested (n)	33

* Seven patients showed biochemical recurrence and thirteen patients showed no biochemical recurrence during follow up.

# One patient was removed from the mean calculation since we were unable to estimate its ancestry accurately due to low concentraton of genomic DNA.

Supplementary Table 2. **Summary of clinical and ancestry data for case-control plasma cohort**

Variable	African American		European American	
	PCa Cases (n =52)	Controls (n = 25)	PCa Cases (n =51)	Controls (n = 23)
Age, ŷ	61.7 ± 9.2	65.2 ± 5.9	63.3 ± 8.9	66.6 ± 9.2
BMI, ŷ	28.1 ± 4.7	29.9 ± 5.1	27.1 ± 3.5	27.5 ± 4.5
Smoking status	Never: 19	Never: 10	Never: 21	Never: 10
	Ever: 33	Ever: 15	Ever: 30	Ever: 13
Gleason Grade	Low (≤6): 24	-	Low (≤6): 28	-
	High (≥7): 28	-	High (≥7):23	-
Recurrence	11*		3*	
PSA(ng/ml), ŷ	7.0 ± 3.6[#]	-	7.8 ± 5.3	-
West African, ŷ	0.6 ± 0.3	0.7 ± 0.1	0.3 ± 0.3[$]	0.1 ± 0.0
European, ŷ	0.3 ± 0.3	0.3 ± 0.1	0.7 ± 0.3	0.9 ± 0.0
Native American, ŷ	0.1 ± 0.0	0.0 ± 0.0	0.0 ± 0.0	0.0 ± 0.0
Genetic Ancestry tested (n)	66		58	

*Eleven AA and three EA patients showed biochemical recurrence. The recurrence information of other patients remains unknown.

#Five AA PCa patients were removed from the mean calculation since their PSA values were >40 ng/ml

$ Three European American PCa cases had West African ancestry >0.74 (which is the median West African ancestry value in AA cases and controls).

Supplementary Table 3. Summary of clinical and ancestry data for case-control urine cohort

Variable	African American		European American	
	PCa Cases (n =20)	Controls (n = 19)	PCa Cases (n =18)	Controls (n = 19)
Age, ŷ	62.5 ± 7.7	65.3 ± 8.9	62.5 ± 10.0	70.9 ± 9.9
BMI, ŷ	27.9 ± 5.0	29.9 ± 5.3	28.1 ± 3.0	28.1 ± 4.6
Gleason Grade	Low (≤6): 7	-	Low (≤6): 8	-
	High (≥7): 13	-	High (≥7):10	-
Recurrence	7*		1	
PSA(ng/ml), ŷ	9.8 ± 8.6#	-	9.7 ± 9.3	-
West African, ŷ*	0.8 ± 0.1	0.8 ± 0.1	0.1 ± 0.1	0.1 ± 0.0
European, ŷ*	0.1 ± 0.1	0.2 ± 0.1	0.8 ± 0.1	0.9 ± 0.0
Native American, ŷ	0.1 ± 0.1	0.0 ± 0.0	0.1 ± 0.1	0.0 ± 0.0
Genetic Ancestry Verified (n)	27		31	

*Seven AA and one EA patients showed biochemical recurrence. The recurrence information of other patients remains unknown.

One AA PCa case was removed from the mean PSA calculation since he had PSA>40 ng/ml.

Supplementary Table 4. Summary of clinical and ancestry data for the biopsy plasma cohort

Variable	African American	
	Biopsy Positive (n =80)	Biopsy Negative (n = 43)
Age, ŷ	63.6 ± 5.9	63.8 ± 7.0
BMI, ŷ	29.0 ± 4.7	29.9 ± 5.3
Smoking status	Never: 20	Never: 10
	Ever: 60	Ever: 33
Gleason Grade	Low (≤6): 21	-
	High (≥7): 59	-
Recurrence	8*	
PSA(ng/ml), ŷ	7.9 ± 4.5#	6.9 ± 3.4
West African, ŷ	0.8 ± 0.1	0.8 ± 0.0
European, ŷ	0.2 ± 0.1	0.2 ± 0.1
Native American, ŷ	0.0 ± 0.0	0.0 ± 0.0
Genetic Ancestry Verified (n)	123	

At the end of two years of clinical follow up, two biospy negative controls became biopsy positive

*Eight patients showed biochemical recurrence and 47 patients showed no biochemical recurrence.

#Six biopsy postive patients removed from the mean PSA calculation since they had PSA>40 ng/ml.

Supplementary Table 5. List of precursor and product ion transitions of internal standards used for Multiple Reaction Monitoring (MRM) mass spectrometry.

Internal Standards used for MRM experiments:

Platforms*	Compound Name	Precursor Ion m/z	Product Ions m/z	Polarity	RT(min)
1	Arginine ^{15}N2	177.1	159.1/116.1/70.1	Positive	6.6
1	Jasmonic acid	211.1	151.1/133.1/118.9	Positive	3.8
1	Thymine D4	131.1	113.9	Positive	7.3
1	Tryptophan ^{15}N2	207.1	189/147.1	Positive	5.8
1	Zeatine	220	136/119	Positive	5.7
2	Gibberllic acid	345	143	Negative	5.4
2	Anthranilic acid ^{15}N	137	93	Negative	6.6
2	Gibberllic acid	345	143	Negative	4.9
2	Jasmonic acid	209	126.9/59	Negative	6.6
2	Threonic acid	135	75.1	Negative	6.5
2	Tryptophan ^{15}N2	205	117	Negative	3.2
2	Zeatine	218	133.5/173	Negative	2.5
3	Creatinine ^{13}C	115	98.9/87.1/45.1	Positive	3.5
3	Tryptophan ^{15}N2	207.1	189/147.1	Positive	3
3	Zeatine	220	136/119	Positive	2.4
4	Gibberllic acid	345	143	Negative	4.9
4	Glutamic acid D5	151	107	Negative	6.1
4	Jasmonic acid	209	126.9/59	Negative	3.4
4	Tryptophan ^{15}N5	205	117	Negative	3.2
4	Zeatine	218	133.5/173	Negative	2.5

*Four chromatography platforms were used to separate all the metabolites in the study: Platform 1: column, Amide XBridge(Waters); mobile phase, A: 0.1% formic acid in HPLC grade water,B: 0.1% formic acid in HPLC grade acetonitrile.

Platform 2: column, Amide XBridge(Waters); mobile phase, A: 20mM ammonium acetate in HPLC grade water pH 9,B: HPLC grade acetonitrile.

Platform 3: column, Luna NH2(Waters); mobile phase, A: 20mM ammonium acetate in HPLC grade water pH 9,B: HPLC grade acetonitrile.

Platform 4: column, Luna NH2(Waters); mobile phase, A: 20mM ammonium acetate in HPLC grade water pH 9,B: HPLC grade acetonitrile.
Platform 1 and platform 3 were used in positive polarity and Platform 2 and Platform 4 were used in negative polarity.

Supplementary Table 6. List of all metabolites measured in tissues and their corresponding MRM transitions:

Platforms	Compound Name	RT(min)	Precursor Ion	Product Ions	Polarity
2	1,3-Bisphospho-D-glycerate(1,3-BPG)	4.1	265	79	Neg
1	1-Methyl tryptophan	5.4	219	202.1/132.1	Pos
1	1-Methyl-Histidine	6.2	170	124	Pos
1	1-Methylnicotinamide	5.7	138	95.1/79.1	Pos
1	1-Stearoyl-sn-glycero-3-phosphocholine (18:0 Lyso PC)	4.4	524	184.1/104.1	Pos
2	2,3-Dihydroxybenzoic acid	3.8	153	109	Neg
1	2-Aminoheptanoic acid	6.3	146	100	Pos
1	2-Aminooctanoic acid	5	160	55	Pos
2	2'-Deoxyguanosine 5'-diphosphate (dGDP)	6.8	426	159	Neg
1	2-Hydroxypyridine	5.3	96	51.1/78.1	Pos
3	2-Methylbutyrylcarnitine	2.4	246	187	Pos
1	2-Methylglutamate	5.9	162	116	Pos
1	2-Methylglutarate	6.3	147	59/55	Pos
1	3-Hydroxy-3-methylglutaryl-CoA (HMG-CoA)	6.7	912	428/136/405.2	Pos
1	3-Hydroxykynurenine	6.5	225	162	Pos
2	3-Phosphoglycerate(3PG)	5.8	185	97	Neg
1	3-Phosphoserine	6.5	186	88	Pos
1	4 Hydroxybutyric acid (GHB)	5.7	105	45/60.1	Pos
1	4-Acetamidobutanoate	5.2	146	86	Pos
1	4-Aminobutyraldehyde	6.4	88	70	Pos
1	4-Aminobutyrate	5.6	104	44.1/69	Pos
1	4-Methyl-2-oxopentanoate	6.7	131	43.06/71	Pos
1	5-Aminoimidazole-4-carboxamide ribotide (AICAR)	6.8	339	110	Pos
1	5-Hydroxyindoleacetate (5-HIAA)	5.7	192	146/91	Pos
1	5-Oxoproline	6.4	130	84	Pos
2	6-Phospho-D-gluconate(6P-gluconate)	6.9	275	97	Neg
1	7-methylguanosine	6.3	299	166	Pos
2	Acetyl phosphate	6.8	139	79	Neg
1	Acetylcarnitine	5.3	204	85	Pos
1	Acetylcholine	5.4	146	43	Pos
1	Adenine	6.8	137	119	Pos

3	Adenosine	2.5	268	136	Pos
1	Adenosine 3,5-cyclic monophosphate (cAMP)	6.4	330	136/119	Pos
1	Adenosine 5-monophosphate (AMP)	6.8	348	136/97	Pos
1	Adenosine diphosphate ribose(ADR)	7.2	560	136.1/348.1	Pos
2	ADP	6.8	426	159	Neg
1	Allo-Threonine	6.3	120	74.1/56.1	Pos
3	Amino Adipic acid	5.9	162	55.1/98.1	Pos
3	Aminophosphovaleric acid (AP5)	2.6	132	86	Pos
2	Anthranilate	5.8	136	92	Neg
1	Arginine	6.3	175	60.2/70.1	Pos
1	Argininosuccinate	5.5	291	70	Pos
1	Asparagine	6.6	133	87.1/74	Pos
1	Aspartame	6.4	295	180.1/120.1	Pos
1	Aspartate-phenyalanine(ASP-PH)	5.5	281	166.1/120.1/88	Pos
3	Aspartic acid	2.2	134	73.9/116	Pos
3	Betaine	2.9	118	59.2/58.1	Pos
3	Betaine aldehyde	1.7	102	58	Pos
2	Butyrylcarnitine	4.3	232	173/86	Pos
4	Carbamoyl phosphate	10.3	140	79	Neg
1	Carnitine	5.6	162	60.1/103	Pos
2	Cholesterol sulfate	3.3	465	97	Neg
2	Choline	5.7	105	45/60.2	Pos
4	Citrate	14.3	191	111/87	Neg
1	Citrulline	6.5	176	159/70.1	Pos
3	Creatine	1.3	132	90/44.1	Pos
1	Creatinine	5.8	114	90/44/86.2	Pos
1	Cystathionine	6.7	223	134.1/88.1	Pos
1	Cysteine	6.8	122	59/76	Pos
1	Cystine	6.9	241	74.1/152	Pos
1	Cytidine 5 -Monophosphate (5-CMP)	6.8	324	112/83.6	Pos
1	Cytosine	6	112	95	Pos
1	Deoxycarnitine	6.5	146	60.1/87.1	Pos
1	Deoxycytidine monophosphate (dCMP)	6.3	308	112	Pos
1	Deoxyguanosine monophosphate (dGMP)	6.8	348	135	Pos
4	D-Gluconic acid	5.9	195	129.1/75.1	Neg
1	D-Glucosamine 1-phosphate	3.8	260	162	Pos

4	Dihomo-gamma-linolenic acid	3.2	305	59	Neg
2	Dihydroorotate	6.2	157	113	Neg
1	Dimethylarginine	6.2	203	70.1/46.1	Pos
1	DL-Pipecolic acid	6.3	130	84	Pos
1	Ergothioneine	6.3	230	186.1059/ 127.0	Pos
1	Erythrono-1,4-lactone	5.9	119	91.1/60	Pos
2	Farnesyl diphosphate	5.4	381	78.7/189.7	Neg
4	Fructose	3.2	179	59	Neg
2	Fructose 6-phosphate(Fructose6P)	6.5	259	97.1/79.1	Neg
2	Fumaric acid	6.3	115	71.1/27.2	Neg
4	Geranyl diphosphate	14.8	313	78.7/155.7	Neg
1	Glucosamine	6.5	180	162/72.1	Pos
5	Glucose	3.2	179	59	Neg
3	Glucose 6-phophate(Glucose6P)	6.5	259	97.1/79.2	Neg
2	Glucuronic acid	6.6	193	113/73.1	Neg
1	Glutamic acid	6.2	148	84/56	Pos
1	Glutamine	6.9	147	130.1/84.1	Pos
1	Glutamyl alanine	6.4	219	84.1/44.1	Pos
2	Glutathione disulfide	6.8	611	306	Neg
1	Glutathione, Reduced (GSH)	6.1	308	76/179	Pos
1	Glycerol 3-phosphate(Glycerol 3P)	6.6	173	132/99	Pos
3	Glycerophosphocholine(GPC)	3.7	258	104	Pos
1	Glycine	6.2	76	31	Pos
1	Glycine-Glycine(Gly-Gly)	5.7	133	30/76.04/4 2.03	Pos
1	Gly-Leu	6.2	189	171/130	Pos
1	GMP	7	364	152/135	Pos
3	Guanidinoacetic acid	3.1	118	30.1/43.1	Pos
1	Guanosine	6.1	284	135/152	Pos
1	Heptanoyl carnitine	7	260	201.2/85	Pos
2	Hexose-6 Phosphate	6.5	259	79	Neg
1	Histamine	6.7	112	95.1/41.1	Pos
1	Histidine	6.9	156	83/110.1	Pos
1	Homocysteine	6.1	136	90/56.1	Pos
2	Homoserine	6.3	120	74.1/56.2	Pos
3	Homovanillic acid	3.4	183	137	Pos
4	Hydroxy glutarate	6.1	147	129.1/101. 1	Neg
2	Hydroxyisocaproic acid(HICA)	5.4	131	85	Neg
1	Hypoxanthine	5.7	137	119/55.1	Pos
3	Imidazole	1.8	69	42	Pos

3	Indole	2.6	118	91	Pos
2	Inosine	6	267	135	Neg
1	Inositol 1-phosphate(Inositol 1P)	6.7	261	234.9/158	Pos
1	Isobutyryl Carnitine	4.3	232	173/85	Pos
1	Isoleucine	5.6	132	43.9/86.1	Pos
1	Isovalerylcarnitine	6.6	246	187.1/85	Pos
2	Ketoglutarate	6.7	145	101.1/57.1	Neg
1	Kynurenic acid	5.5	190	144.1/89	Pos
1	Kynurenine	5.7	209	192/146	Pos
2	Lactate	6.2	89	43	Neg
1	Lysine	6.3	147	84.2/130.2	Pos
2	Malic acid	6.5	133	115.1/71.1	Neg
2	Malonate	6.5	103	59.1/41.1	Neg
1	Malonyl-CoA	5.8	854	347/303/428	Pos
2	Maltose	6.6	341	161/73	Neg
2	Maltotetraose	7	665	161	Neg
4	Mannose	3	179	89.1/71.1	Neg
3	Methionine	3.2	150	56.1/61.1	Pos
2	Methyl alanine	5.4	104	58.1/45	Pos
3	Methylcysteine	2.4	136	119	Pos
4	Methylmalonic acid	14	117	73	Neg
1	Myo-Inositol	6.6	181	139.9/105.2	Pos
1	Myristic acid	7	229	39	Pos
1	Myristoleic acid	3.5	227	43.1/41.2	Pos
1	N, N Dimethyl Glycine	5.4	104	58.1/44	Pos
2	N-Acetyl Aspartic acid	6.4	174	88	Neg
1	N-Acetyl Lysine	6.1	189	129/84.1	Pos
1	N-Acetyl Methionine	5.7	192	104.1/56.1	Pos
4	N-acetyl serine	5.6	146	98/84.1	Neg
1	N-acetylalanine	5.9	132	90/44.1	Pos
1	N-Acetylaspartylglutamate(NAAG)	5.6	305	148.2/84.1	Pos
3	N-Acetylglucosamine	2.4	222	138.1/125.9	Pos
1	N-acetyl-glutamine	6.2	222	171/130	Pos
1	N-Acetylornithine	6.2	175	70.1/115	Pos
1	N-carbamoyl-L-aspartate	6.2	177	74	Pos
1	Nicotinamide	5.3	123	80.1/78	Pos
1	Nicotinamide adenine dinucleotide (NAD)	6.9	664	428/136	Pos
1	Nicotinamide ribonucleotide	6.6	335	123	Pos

1	Nonadecanoic acid	3.3	297	281	Pos
1	Octanoylcarnitine	4.3	288	85/57.1	Pos
2	Octulose-monophosphate (O8P-O1P)	6.8	319	97	Neg
2	P-aminobenzoate	5.8	136	92	Neg
3	Pantothenic acid	4.8	220	90	Pos
3	P-coumaric acid	3.5	165	147/91.1	Pos
1	Phenylalanine	5.6	166	103.1/120.1	Pos
1	Phenylalanine-Glutamate(Phe-Glu)	6.5	295	120/136	Pos
1	Phospho Choline	6.5	185	99/87.1	Pos
2	Phosphoenolpyruvate(PEP)	6.3	167	79/63	Neg
1	Proline	6.2	116	70.1/43	Pos
3	Propeonyl Carnitine	2.8	218	85/60.1	Pos
1	Purine	5.6	121	94	Pos
1	Putrescine	7.1	89	72.1/30	Pos
4	Pyruvate	3.2	87	43/41	Neg
3	Riboflavin	2.3	377	243/172	Pos
4	Ribose	5	149	65/59.1	Neg
2	Ribose-5-phosphate	5.4	229	138.98/96.97	Neg
1	Sacosine	6.3	90	72.1/44.1	Pos
1	Alanine	6.3	90	72.1/44.1	Pos
1	S-Adenosyl-L-homocysteine(SAH)	6.7	385	136.1/134	Pos
3	S-Adenosyl-L-methionine (SAM)	3.5	399	250.1/136.1	Pos
2	Sedoheptulose-1,7-phosphate(sedoheptulose-1,7P)	6.8	289	97	Neg
1	Serine	6.4	106	88.1/60.1	Pos
2	Shikimate	3.8	173	93	Neg
4	Shikimate-3-phosphate	14.2	253	97	Neg
3	S-methyl-5-thioadenosine(MTA)	2	298	136	Pos
2	sn-glycerol-3-phosphate	5.8	171	79	Neg
1	Spermidine	7.5	146	112.1/72.1	Pos
1	S-ribosyl-L-homocysteine	6.6	268	88	Pos
2	Succinate	6.4	117	73/99.9	Neg
1	Succinyl-CoA	5.9	868	428/361/259	Pos
2	Taurine	6.3	124	80	Neg
1	Thiamine	6.8	266	122	Pos
1	Tryptophan	5.8	205	188.1/146.1	Pos
1	Tyrosine	6.1	182	91/136.1	Pos

2	UDP-D-glucose	6.5	565	323	Neg
2	UDP-D-glucuronate	6.5	579	403	Neg
2	UDP-N-acetyl-glucosamine	6.4	606	385	Neg
1	Uracil	6.6	113	72/68.1	Pos
1	Urea	5.6	61	44	Pos
4	Uric acid	8.8	167	124	Neg
2	Uridine	5.8	243	200	Neg
1	Uridine 5'-monophosphate (UMP)	6.8	325	113/97	Pos
1	Valine	5.9	118	55/72.2	Pos
3	Xanthine	1.8	153	136/110	Pos

*There are four chromatography platforms used to separate all the metabolites in the study: Platform 1: column, Amide XBridge(Waters); mobile phase, A: 0.1% formic acid in HPLC grade water,B: 0.1% formic acid in HPLC grade acetonitrile.

Platform 2: column, Amide XBridge(Waters); mobile phase, A: 20mM ammonium acetate in HPLC grade water pH 9,B:HPLC grade acetonitrile.

Platform 3: column, Luna NH2(Waters); mobile phase, A: 20mM ammonium acetate in HPLC grade water pH 9,B: HPLC grade acetonitrile.

Platform 4: column, Luna NH2(Waters); mobile phase, A: 20mM ammonium acetate in HPLC grade water pH 9,B: HPLC grade acetonitrile.

Platform 1 and platform 3 were used in positive polarity and Platform 2 and Platform 4 were used in negative polarity.

Supplementary Table 7. The percent coefficient of variation (% CV) of detected metabolites forthe liver pool

Metabolite	CV(%)
1,3-BPG	5.76%
18:0 Lyso PC	12.23%
1-Methyl tryptophan	2.97%
1-Methyl-Histidine	4.56%
1-Methylnicotinamide	2.59%
2,3-DHBA	5.97%
2-Aminoheptanoic acid	1.05%
2-Aminooctanoic acid	9.55%
2-Hydroxypyridine	5.67%
2-Methylbutyroylcarnitine	5.15%
2-Oxoisocaproate	3.79%
3-OH-kynurenine	7.63%
3PG	4.87%
3-Phosphoserine	1.77%
4-Acetamidobutanoate	3.47%
4-aminobutanal	7.64%
4-Aminobutyrate	3.22%
5-HIAA	1.55%
5-Oxoproline	1.46%
6P-gluconate	3.26%
7-methylguanosine	2.88%
Acetyl Aspartic acid	23.02%
Acetyl Lysine	1.75%
Acetyl Methionine	1.66%
Acetyl phosphate	3.84%
Acetyl serine	13.70%
Acetylalanine	1.65%
Acetylcarnitine	5.22%
Acetylcholine	5.57%
Acetylglucosamine	5.05%
Acetyl-glutamine	5.83%
Acetylornithine	3.29%
Adenine	7.63%
Adenosine	2.36%
ADP	5.79%
ADR	14.55%
AICAR	7.95%

Aminoadipic acid	3.94%
Aminobenzoate	7.27%
AMP	2.85%
Anthranilate	2.72%
AP5	2.80%
Arginine	1.67%
Argininosuccinate	2.34%
Asparagine	7.64%
Aspartame	1.91%
Aspartic acid	2.02%
ASP-PH	5.12%
Betaine	11.52%
Betaine aldehyde	10.12%
Butanoylcarnitine	3.16%
Carbamoyl-L-aspartate	5.38%
Carbamoyl-P	1.13%
Carnitine	4.57%
Cholesterol sulfate	2.97%
Choline	8.99%
Citrate	9.68%
Citrulline	6.72%
CMP	12.44%
Creatine	3.67%
Creatinine	4.15%
cyclic AMP	15.36%
Cystathionine	4.31%
Cysteine	7.93%
Cystine	8.98%
Cytosine	5.08%
dCMP	2.59%
Deoxycarnitine	6.21%
dGDP	3.95%
DGLA	2.97%
dGMP	3.63%
Dihydroorotate	4.09%
Dimethylarginine	11.79%
Ergothioneine	2.76%
Erythrono-1,4-lactone	4.71%
Farnesyl diphosphate	7.35%
Fructose	2.29%
Fructose 6-P	2.51%

Fumaric acid	1.58%
Geranyl diphosphate	2.79%
GHB	4.60%
Gluconic acid	12.06%
Glucosamine	4.79%
Glucosamine 1P	8.04%
Glucose	9.41%
Glucose 6-P	12.15%
Glucuronic acid	8.89%
Glutamate	2.93%
Glutamine	10.16%
Glutamyl alanine	1.26%
Glutathione disulfide	11.73%
Glycerol 3P	2.46%
Glycerol-3-P	3.53%
Glycine	2.86%
Gly-Gly	1.88%
Gly-Leu	1.39%
GMP	1.55%
GPC	2.57%
GSH	13.25%
Guanidinoacetic acid	6.52%
Guanosine	5.23%
Heptanoyl carnitine	3.12%
Hexose-P	6.13%
HICA	19.13%
Histamine	7.57%
Histidine	7.92%
HMG-CoA	4.00%
Homocysteine	8.59%
Homoserine	2.58%
Homovanillic acid	1.53%
Hypoxanthine	2.65%
Imidazole	1.56%
Indole	6.20%
Inosine	1.56%
Inositol 1-P	1.70%
Isoleucine	2.44%
Isovalerylcarnitine	4.99%
Ketoglutarate	9.22%
Kynurenic acid	4.45%

Kynurenine	11.75%
Lactate	2.26%
Lysine	6.19%
Malic acid	3.75%
Malonate	2.11%
Malonyl-CoA	6.80%
Maltose	2.01%
Maltotetraose	2.04%
Mannose	8.18%
Methionine	5.76%
Methyl alanine	3.47%
Methylcysteine	1.39%
methyl-Glu	2.42%
Methylglutaric acid	4.78%
Methylmalonic acid	5.57%
MTA	1.53%
Myo-Inositol	8.43%
Myristate	2.43%
Myristoleic acid	5.90%
N, N Dimethyl Glycine	4.62%
NAAG	5.85%
NAD	2.91%
Nicotinamide	11.83%
Nicotinamide ribotide	1.61%
Nonadecanoic acid	1.79%
O8P-O1P	11.09%
Octanoylcarnitine	4.05%
OH-Glutarate	2.26%
Pantothenic acid	1.74%
P-coumaric acid	5.05%
PEP	3.06%
Phe-Glu	6.13%
Phenylalanine	6.14%
Phospho Choline	2.76%
Pipecolic acid	9.72%
Proline	7.39%
Propeonyl Carnitine	2.45%
Purine	3.25%
Putrescine	13.34%
Pyruvate	3.53%
Riboflavin	4.92%

Ribose	2.51%
Ribose-5P	3.50%
Sacosine/Alanine	4.19%
SAH	1.97%
SAM	3.76%
Sarcosine/Alanine	3.22%
Sedoheptulose-1,7P	2.20%
Serine	17.70%
Shikimate	2.92%
Shikimate-3P	6.02%
Spermidine	2.22%
SRH	10.57%
Succinate	13.38%
Succinyl-CoA	4.53%
Taurine	3.14%
Thiamine	3.16%
Threonine	4.43%
Tryptophan	5.72%
Tyrosine	2.44%
UDP-GlcNAc	4.42%
UDP-glucose	2.92%
UDP-glucuronate	2.25%
UMP	13.98%
Uracil	3.10%
Urea	6.65%
Uric acid	4.25%
Uridine	3.35%
Valine	4.16%
Xanthine	3.00%

Supplementary Table 8. List of metabolites alerted between AA PCa and matched benign adjacent tissues (FDR≤ 0.26 by Benjamin Hochberg Method). Raw P values are also provided.

Metabolite	P-Value	FDR	Fold Change
Glycine-Leucine (Gly-Leu)	0.000	0.003	2.604
Cystathionine	0.002	0.024	2.593
Acetyl-glutamine	0.000	0.003	2.561
Cystine	0.052	0.183	2.263
Acetyl Lysine	0.000	0.003	2.150
Nicotinamide ribnucleotide	0.035	0.139	2.088
7-methylguanosine	0.008	0.046	1.973
Riboflavin	0.003	0.027	1.756
Heptanoyl-carnitine	0.002	0.027	1.734
Methyl-Glutamate	0.000	0.004	1.732
Hypoxanthine	0.004	0.028	1.728
Phenylalanine-Glutamate (Phe-Glu)	0.003	0.028	1.714
Proline	0.000	0.004	1.676
Methionine	0.000	0.003	1.653
Homocysteine	0.007	0.041	1.651
Glycerophosphocholine (GPC)	0.004	0.031	1.637
Ergothioneine	0.012	0.062	1.612
Guanosine monophosphate (GMP)	0.032	0.134	1.522
Methyl alanine	0.005	0.031	1.481
N, N Dimethyl Glycine	0.005	0.031	1.481
P-coumaric acid	0.000	0.003	1.466
4 Hydroxybutyric acid (GHB)	0.004	0.028	1.463
Choline	0.004	0.028	1.463
Aminoadipic acid	0.030	0.131	1.446
Tyrosine	0.005	0.032	1.437
Succinyl-CoA	0.043	0.160	1.432
Citrulline	0.002	0.023	1.413
Serine	0.091	0.252	1.404
Glycine	0.005	0.032	1.403
Pantothenic acid	0.012	0.062	1.401
Guanosine	0.039	0.151	1.400
2,3-Dihydroxybenzoic acid (2,3-DHBA)	0.094	0.252	1.392
Adenosine 3,5-cyclic monophosphate (cAMP)	0.029	0.127	1.391
Valine	0.001	0.019	1.386
1-Methyl-Histidine	0.001	0.019	1.384
Sarcosine/Alanine	0.013	0.065	1.384
4-Aminobutanal	0.006	0.035	1.366

2-Aminoheptanoic acid	0.008	0.045	1.362
Homoserine	0.001	0.019	1.356
Threonine	0.001	0.019	1.356
S-ribosyl-L-homocysteine (SRH)	0.035	0.139	1.335
1-Methylnicotinamide	0.092	0.252	1.333
Aminobenzoate	0.061	0.198	1.317
Glutamine	0.001	0.022	1.315
Arginine	0.028	0.124	1.314
Acetylornithine	0.028	0.124	1.314
S-Adenosyl-L-homocysteine (SAH)	0.020	0.095	1.313
Lysine	0.003	0.028	1.303
Anthranilate	0.072	0.225	1.302
5-Oxoproline	0.002	0.023	1.297
Malic acid	0.003	0.028	1.277
Glutamate	0.003	0.028	1.273
Kynurenine	0.055	0.189	1.269
Pipecolic acid	0.002	0.027	1.267
Methylglutaric acid	0.009	0.046	1.267
Histidine	0.017	0.085	1.260
Phenylalanine	0.043	0.160	1.258
Carnitine	0.049	0.179	1.237
Glutamyl alanine	0.076	0.233	1.221
Guanidinoacetic acid	0.068	0.215	1.220
Aminophosphovaleric acid (AP5)	0.035	0.139	1.220
Isoleucine	0.081	0.238	1.202
Adenosien 5-monophosphate (AMP)	0.079	0.238	1.190
Fumaric acid	0.060	0.198	1.187
Hydroxyisocaproic acid (HICA)	0.097	0.255	1.136
Adenosine diphosphate (ADP)	0.093	0.252	0.814
2'-Deoxyguanosine 5'-diphosphate (dGDP)	0.096	0.255	0.813
3-Phosphoglycerate (3PG)	0.051	0.180	0.808
Betaine aldehyde	0.068	0.215	0.801
Hexose-Phosphate	0.100	0.259	0.783
Fructose 6-Phosphate	0.084	0.241	0.774
Argininosuccinate	0.082	0.240	0.765
Glucose 6-Phosphate	0.057	0.191	0.735

Supplemental Table 9. List of enriched pathways obtained with Network-based gene set enrichment analysis (**NetGSA**) of 190 metabolites **detected in** AA PCa and matched adjacent benign tissues(p value **in this table** was calculated and q value was computed by Benjamin Hochberg method. Network representation of concept enriched at q value <1E-05 is shown in Figure 1B). **190 metabolites mapped to a total of 197/236 KEGG pathways corresponding to a coverage of ~83.5 %**

Pathway/Metabolism	q Value	Direction
Adherens junction	5.2E-12	UP IN CANCER
Rheumatoid arthritis	5.2E-12	UP IN CANCER
Cysteine and methionine	2.8E-11	UP IN CANCER
Metabolic pathways	5.3E-09	UP IN CANCER
Selenocompound	5.6E-09	UP IN CANCER
Glycine, serine and threonine	7.9E-09	UP IN CANCER
Glycosaminoglycan degradation	3.0E-07	UP IN CANCER
Tyrosine	7.3E-07	UP IN CANCER
Vitamin B6	1.1E-06	UP IN CANCER
Endocrine and other factor-regulated calcium reabsorption	1.8E-06	UP IN CANCER
Pyrimidine	1.9E-06	UP IN CANCER
Glycosphingolipid biosynthesis - ganglio series	5.2E-06	UP IN CANCER
Alcoholism	1.3E-05	UP IN CANCER
Serotonergic synapse	1.3E-05	UP IN CANCER
Chemical carcinogenesis	1.4E-05	UP IN CANCER
Nicotinate and nicotinamide	1.5E-05	UP IN CANCER
Amphetamine addiction	1.5E-05	UP IN CANCER
Cocaine addiction	1.5E-05	UP IN CANCER
Cholinergic synapse	1.6E-05	UP IN CANCER
Porphyrin and chlorophyll	1.8E-05	UP IN CANCER
Phenylalanine	2.4E-04	UP IN CANCER
Lysine degradation	2.4E-04	UP IN CANCER
2-Oxocarboxylic acid	3.0E-04	UP IN CANCER
Other glycan degradation	4.3E-04	UP IN CANCER
Osteoclast differentiation	4.3E-04	UP IN CANCER
Retinol	5.4E-04	UP IN CANCER
Dopaminergic synapse	5.4E-04	UP IN CANCER
One carbon pool by folate	5.7E-04	UP IN CANCER
Alanine, aspartate and glutamate	6.4E-04	UP IN CANCER
Purine	1.3E-03	UP IN CANCER
Drug - other enzymes	1.3E-03	UP IN CANCER
Aminoacyl-tRNA biosynthesis	2.3E-03	UP IN CANCER
Circadian entrainment	2.3E-03	UP IN CANCER
Amyotrophic lateral sclerosis (ALS)	2.3E-03	UP IN CANCER

PPAR signaling pathway	2.3E-03	UP IN CANCER
Tryptophan	2.9E-03	UP IN CANCER
Caffeine	3.4E-03	UP IN CANCER
Fat digestion and absorption	3.9E-03	UP IN CANCER
Melanogenesis	4.4E-03	UP IN CANCER
Ubiquinone and other terpenoid-quinone biosynthesis	5.8E-03	UP IN CANCER
Synthesis and degradation of ketone bodies	6.6E-03	UP IN CANCER
Alzheimer's disease	7.6E-03	UP IN CANCER
Arginine and proline	1.0E-02	UP IN CANCER
Thiamine	1.0E-02	UP IN CANCER
Drug - cytochrome P450	1.0E-02	UP IN CANCER
Dilated cardiomyopathy	1.1E-02	UP IN CANCER
Gastric acid secretion	1.1E-02	UP IN CANCER
Oocyte meiosis	1.1E-02	UP IN CANCER
Vascular smooth muscle contraction	1.1E-02	UP IN CANCER
Vasopressin-regulated water reabsorption	1.1E-02	UP IN CANCER
Vibrio cholerae infection	1.1E-02	UP IN CANCER
Gap junction	1.1E-02	UP IN CANCER
Long-term potentiation	1.1E-02	UP IN CANCER
Retrograde endocannabinoid signaling	1.1E-02	UP IN CANCER
Huntington's disease	1.4E-02	UP IN CANCER
GnRH signaling pathway	1.4E-02	UP IN CANCER
Peroxisome	1.4E-02	UP IN CANCER
Pathways in cancer	1.6E-02	UP IN CANCER
Bladder cancer	2.3E-02	UP IN CANCER
Citrate cycle (TCA cycle)	2.4E-02	UP IN CANCER
Glutamatergic synapse	2.4E-02	UP IN CANCER
Glyoxylate and dicarboxylate	2.5E-02	UP IN CANCER
Valine, leucine and isoleucine degradation	2.5E-02	UP IN CANCER
Long-term depression	3.3E-02	UP IN CANCER
Phenylalanine, tyrosine and tryptophan biosynthesis	4.4E-02	UP IN CANCER
Chagas disease (American trypanosomiasis)	4.4E-02	UP IN CANCER
Renin-angiotensin system	4.4E-02	UP IN CANCER
Glycosphingolipid biosynthesis - globo series	1.1E-06	DOWN IN CANCER
Glycolysis / Gluconeogenesis	2.4E-04	DOWN IN CANCER
Phototransduction	5.1E-04	DOWN IN CANCER
Linoleic acid	2.5E-03	DOWN IN CANCER
Terpenoid backbone biosynthesis	3.1E-03	DOWN IN CANCER

Influenza A	4.1E-03	DOWN IN CANCER
Galactose	5.0E-03	DOWN IN CANCER
p53 signaling pathway	7.2E-03	DOWN IN CANCER
Type II diabetes mellitus	2.8E-02	DOWN IN CANCER
Oxidative phosphorylation	2.8E-02	DOWN IN CANCER
Vitamin digestion and absorption	4.2E-02	DOWN IN CANCER
Pyruvate	4.7E-02	DOWN IN CANCER

Supplementary Table 10. Results of multivariate analysis for Meat Doneness using linear regression in Cancer Cases and controls to determine its association with plasma methionine and homocysteine levels.

	Methionine			Homocysteine			Methionine			Homocysteine		
Variable	Parameter Estimate	P value		Parameter Estimate	P value	Adjusted R^2	Parameter Estimate	P value	Adjusted R^2	Parameter Estimate	P value	Adjusted R^2
Diagnosis	Cancer	Cancer	Cancer	Cancer	Cancer	Cancer	Control	Control	Control	Control	Control	Control
Race	0.300	0.004	0.190	4.80	1.3E-08	0.65	0.245	0.040	0.070	0.014	0.96	-0.053
Age at diagnosis	0.003	0.504	0.190	-0.06	0.110	0.65	-0.007	0.340	0.070	0.008	0.663	-0.053
BMI	-0.006	0.506	0.190	0.15	0.020	0.65	0.001	0.890	0.070	-0.002	0.951	-0.053
Education	0.030	0.557	0.190	0.14	0.740	0.65	0.168	0.070	0.070	0.124	0.606	-0.053
Income	0.090	0.142	0.190	-0.39	0.370	0.65	-0.178	0.010	0.070	-0.270	0.091	-0.053
Number of indivuals in the house hold	-0.030	0.575	0.190	0.48	0.260	0.65	-0.083	0.310	0.070	0.192	0.398	-0.053
Meat Doneness	-0.060	0.529	0.190	1.58	0.030	0.65	-0.133	0.210	0.070	-0.1	0.668	-0.053

Race, age at diagnosis, BMI, education, income and number of individuals in the house hold were controlled in the analysis. Dichotomization schema: Race: EA vs AA; Age at diagnosis: continuous values; BMI: continuous values; Education: Group 1 (Elementary to High School), Group 2: (College and Technical School) and Group 3: (Professional School); Income: Group 1:(<$10,000-$29,000), Group 2: ($ 30,000-$ 59,999), and Group 3: ($ 60,000 and above); Number of individuals in the house hold: Group 1: (1), Group 2: (2) and Group 3: (≥ 3). For stratification of dietary variables, please refer to Supplemental Methods.

Supplementary Table 11. Results of multivariate analysis for fish consumption using linear regression in Cancer Cases and controls to determine its association with plasma methionine and homocysteine levels.

	Methionine			Homocysteine			Methionine			Homocysteine		
Variable	Parameter Estimate	P value	Adjusted R^2	Parameter Estimate	P value	Adjusted R^2	Parameter Estimate	P value	Adjusted R^2	Parameter Estimate	P value	Adjusted R^2
Diagnosis	Cancer	Cancer	Cancer	Cancer	Cancer	Cancer	Control	Control	Control	Control	Control	Control
Race	0.339	0.0003	0.191	4.080	1.5E-07	0.602	0.202	0.080	0.034	0.020	0.96	-0.05
Age at diagnosis	0.003	0.620	0.191	-0.055	0.160	0.602	-0.006	0.440	0.034	0.010	0.510	-0.05
BMI	-0.006	0.520	0.191	0.141	0.050	0.602	-0.001	0.920	0.034	-0.002	0.940	-0.05
Education	0.021	0.710	0.191	0.47	0.290	0.602	0.121	0.180	0.034	0.104	0.650	-0.05
Income	0.079	0.190	0.191	-0.20	0.660	0.602	-0.163	0.040	0.034	-0.283	0.080	-0.05
Number of indivuals in the house hold	-0.051	0.410	0.191	0.71	0.130	0.602	-0.065	0.420	0.034	0.212	0.340	-0.05
Fish Consumption	0.068	0.480	0.191	-0.4	0.570	0.602	-0.059	0.570	0.034	0.1	0.720	-0.05

Race, age at diagnosis, BMI, education, income and number of individuals in the house hold were controlled in the analysis. Dichotomization schema: Race: EA vs AA; Age at diagnosis: continuous values; BMI: continuous values; Education: Group 1 (Elementary to High School), Group 2: (College and Technical School) and Group 3: (Professional School); Income: Group 1:(<$10,000-$29,000), Group 2: ($ 30,000-$ 59,999), and Group 3: ($ 60,000 and above); Number of individuals in the house hold: Group 1: (1), Group 2: (2) and Group 3: (≥ 3). For stratification of dietary variables, please refer to Supplemental Methods.

Supplementary Table 12. Results of multivariate analysis for consumption of bacon fat using linear regression in Cancer Cases and controls to determine its association with plasma methionine and homocysteine levels.

	Methionine			Homocysteine			Methionine			Homocysteine		
Variable	Parameter Estimate	P value	Adjusted R^2	Parameter Estimate	P value	Adjusted R^2	Parameter Estimate	P value	Adjusted R^2	Parameter Estimate	P value	Adjusted R^2
Diagnosis	Cancer	Cancer	Cancer	Cancer	Cancer	Cancer	Control	Control	Control	Control	Control	Control
Race	0.340	0.001	0.215	4.460	1.7E-07	0.638	0.220	0.110	0.087	0.470	0.16	0.002
Age at diagnosis	0.002	0.760	0.215	-0.070	0.103	0.638	-0.009	0.290	0.087	-0.014	0.530	0.002
BMI	-0.009	0.370	0.215	0.18	0.020	0.638	0.008	0.450	0.087	-0.010	0.690	0.002
Education	0.012	0.830	0.215	0.63	0.170	0.638	0.140	0.180	0.087	0.130	0.620	0.002
Income	0.089	0.140	0.215	-0.23	0.610	0.638	-0.141	0.080	0.087	-0.265	0.096	0.002
Number of indivuals in the house hold	-0.068	0.270	0.215	0.592	0.210	0.638	-0.182	0.110	0.087	-0.332	0.280	0.002
Consumption of Bacon Fat	-0.198	0.140	0.215	-0.4	0.700	0.638	-0.047	0.720	0.087	0.1	0.790	0.002

Race, age at diagnosis, BMI, education, income and number of individuals in the house hold were controlled in the analysis. Dichotomization schema: Race: EA vs AA; Age at diagnosis: continuous values; BMI: continuous values; Education: Group 1 (Elementary to High School), Group 2: (College and Technical School) and Group 3: (Professional School); Income: Group 1:(<$10,000-$29,000), Group 2: ($ 30,000-$ 59,999), and Group 3: ($ 60,000 and above); Number of individuals in the house hold: Group 1: (1), Group 2: (2) and Group 3: (≥ 3). For stratification of dietary variables, please refer to Supplemental Methods.

Methionine-homocysteine pathway in African-American prostate cancer

Jie H. Gohlke[1,2,*], Stacy M. Lloyd[1*], Sumanta Basu[3*], Vasanta Putluri[1], Shaiju K Vareed[1], Uttam Rasaily[1] , Danthasinghe Waduge Badrajee Piyarathna[1], Hunter Fuentes[4], Thekkelnaycke Rajendiran[5], Tiffany H. Dorsey[6], Chandrashekar R. Ambati[1], Balasubramanyam Karanam[7], Salil Kumar Bhowmik[1], Rick Kittles[8], Stefan Ambs[6], Martha Pritchett Mims[9], Michael Ittmann[10], Jeffrey A Jones[4], Ganesh Palapattu[11], Nagireddy Putluri[1], George Michailidis[12], Arun Sreekumar[1] #

Clinical samples: All clinical samples used in this study were obtained using informed consent and the approval of the Institutional Review Board at the Baylor College of Medicine and collaborating institutions including National Cancer Institute (Study PI: Dr. Stefan Ambs, co-author). Overall, we used three cohorts of clinical specimens in this study. These include 1) prostatectomy tissue samples which were used for metabolomic and tissue microarray analysis (**Figures 1 A and 2 E,F**); obtained from the Baylor College of Medicine Human Tissue Acquisition and Pathology Core of the Dan L. Duncan Cancer Center, 2) case-Control plasma and urine obtained from the NCI-Maryland Prostate Cancer Case-Control Study (**Figures 1C, E, F and H** and Figs. **2A-C**) and 3) biopsy plasma cohort obtained from the Population Sciences Biorepository at Baylor College of Medicine (**Figures 1 D and G**).

Cohort 1: Prostatectomy Tissue samples: Frozen pathologically-verified prostate tissues (PCa and adjacent benign) for metabolomics and tissue microarrays (TMA) were obtained from the Baylor College of Medicine Human Tissue Acquisition and Pathology Core of the Dan L. Duncan Cancer Center. All samples were collected during radical prostatectomy with informed consent and Institutional Review Board approval and stored retrospectively in a de-identified manner in the tumor bank. All tissue samples were reviewed for their tumor content by a GU pathologist prior to the metabolomics or TMA analysis. Tissue obtained from the cancer samples for

metabolomics analysis contained at least 70% tumors, while benign tissues were free of tumors upon pathological examination. A total of 33 AA PCa-benign pairs were used for the metabolic analysis (see **Supplementary Table 1** for clinical data).

The PCa-tissue microarrays used in this study were built by Baylor College of Medicine's Histology and Pathology Core. The TMA's utilized for *BHMT* staining were comprised of 52 AA-matched PCa and 54 adjacent benign tissue sections collected from 2005–2008 and 31 EA-matched PCa and 39 adjacent benign tissue sections collected from 2009–2012, while TMA's for *CBS* staining included 46 AA matched PCa and 49 adjacent benign tissues and 130 EA PCa cases and 132 controls.

<u>Cohort 2: Case-Control plasma and urine samples</u>

An independent set of 151 (103 cases and 48 controls) case-control plasma samples (AA, 52 cases and 25 controls; EA, 51 cases and 23 controls; **Supplementary Table 2**) were obtained from the National Cancer Institute (PI: Ambs) for LC/MS-MS analysis of methionine and homocysteine. 76 samples (39 from AA and 37 from EA) were used for urinary sarcosine measurement by GC/MS (**Supplementary Table 3**).

The study design used to collect samples for the NCI-Maryland Prostate Cancer Case-Control Study and associated metadata, has been published by the Ambs group [1].

Briefly, the case-control population utilized in the current study is a small sub-sample of a larger population established for the NCI-Maryland Prostate Cancer Case-Control Study. This study was initiated in 2005 after Institutional Review Board approval, and recruitment was completed in 2015. The study was designed to evaluate the role of environmental and ancestry-related factors on prostate cancer susceptibility. Participants were principally residents of four counties in Maryland: Anne Arundel, Baltimore City, Baltimore County, and Howard, but also included residents of

Washington DC, and neighboring counties of Pennsylvania, Delaware, and Virginia. Cases were recruited from the Baltimore Veterans Affairs Medical Center and the University of Maryland Medical Center.

Cases were defined as men with a prostate cancer diagnosis within the last two years, prior to recruitment. Additional inclusion criteria included men that were between 40-90 years of age, natural-born citizens of the United States, and sufficient English-language proficiency.

Controls were recruited through the Maryland Department of Motor Vehicle database and were frequency-matched by age and race to cases. Male controls were those with no known history of cancer other than non-melanoma skin cancer. They had an operational residential phone number, were natural-born citizens of United States, and had sufficient English-language proficiency.

Men with a history of radiation therapy or chemotherapy were not eligible for the study. All participants self-reported their race as either African American (AA) or European American (EA). In total, 976 cases were recruited, which includes 489 AA and 487 EA, and 1034 population controls, including 486 AA and 548 EA.

At the time of enrollment, a survey was administered by a trained interviewer utilizing pre-developed script, to ascertain family, medical, and occupational history, as well as socio-economic status, anthropometrics, tobacco use, sexual history, and nutrition. The NCI's Division of Cancer Epidemiology and Genetics Technical Evaluation Committee reviewed the dietary questionnaire before it was included in the study materials. The nutrition questionnaire is included as **Supplemental material** appended to supplementary Methods section. Specifically, our study included 103 cases and 48 controls from the NCI-Maryland Prostate Cancer Case-Control Study. Retrospectively collected plasma samples were used to measure the levels of metabolites and correlate them with existing nutritional and clinical metadata.

Cohort 3: Biopsy plasma cohort:

An additional AA biopsy sample set of 123 samples (80 biopsy positive and 43 biopsy negative) were obtained from Baylor College of Medicine's Population Sciences

Biorepository and used for analyzing the levels of methionine and homocysteine. This is a clinically challenging cohort of patients with majority of them (n= 111) having prostate specific antigen levels (PSA) >4ng/ml who underwent biopsy to verify presence or absence of prostate cancer. These samples were used to measure methionine and homocysteine levels using mass spectrometry. In addition, we also used genomic DNA from these individuals to perform genetic ancestry estimation. **Supplementary Table 4** summarizes the clinical and ancestry estimates for this cohort.

***Genetic ancestry estimation*:** Genomic DNA was isolated from seminal vesicles or non-cancerous tissue as well as from buffy coat or a cheek swab for all plasma samples and genotyped to determine the proportion of European, West African, and Native American genetic ancestry as previously described [2, 3]. In this study, 105 ancestry-informative markers were genotyped to determine the proportion of European, West African, and Native American genetic ancestry.

***Tissue metabolite profiling*:** High-performance liquid chromatography (HPLC) grade acetonitrile, methanol, and water were purchased from Burdick & Jackson (Morristown, NJ). Mass spectrometry-grade formic acid and internal standards (including ^{15}N-anthranilic acid, ^{13}C-creatinine, zeatine, jasmonic acid, gibberelic acid, threonic acid, $^{15}N_2$-tryptophan, D_4-thymine, D_5-glutamic acid and [$^{15}N_2$]arginine) were purchased from Sigma-Aldrich (St. Louis, MO). The mass spectrometer was calibrated using ESI-L Low Concentration Tuning Mix from Agilent Technologies (Santa Clara, CA).

Prostate cancer and normal adjacent tissues were stored at −140 °C until the analysis. For the extraction of the metabolome, 10 mg of tissue was homogenized in a

1:4 ice-cold water:methanol mixture containing an equimolar mixture of 10 standard compounds: ^{15}N-anthranilic acid (1 Da mass difference from endogenous anthranilic acid), ^{13}C-creatinine (1 Da mass difference from endogenous creatinine), zeatine, jasmonic acid, gibberelic acid, threonic acid, $^{15}N_2$-tryptophan (2 Da mass difference from endogenous tryptophan), D_4-thymine (4 Da mass difference from endogenous thymine), D_5-glutamic acid (5 Da mass difference from endogenous glutamic acid) and [$^{15}N_2$]arginine (2 Da mass difference from endogenous arginine). This was followed by the sequential addition of ice-cold chloroform and water (3:1), and the separation of the organic (methanol and chloroform) and aqueous solvents (water:methanol:chloroform:water; 1:4:3:1). The aqueous extract was deproteinized using a 3-KDa molecular filter (Amicon Ultracel-3K membrane, Millipore Corporation, Billerica, MA), and the filtrate containing the metabolites were dried under vacuum (Genevac EZ-2plus, Gardiner, NY). Prior to MS, the dried extract was resuspended in an identical volume of injection solvent composed of water and methanol (50:50) and subjected to liquid chromatography (LC)-MS.

We have used the pooled samples as controls to monitor the reproducibility of the metabolomic mass spectrometry pipeline. These samples were co-extracted with the clinical samples and analyzed multiple times at regular intervals. The data from these pooled samples was used to calculate the CV described in the main text

and shown in **Supplementary Table 7**.

Suspended samples (10 µL) were injected and analyzed using a 6495 triple quadrupole mass spectrometer (Agilent Technologies, Santa Clara, CA) coupled to a 1290 HPLC system (Agilent Technologies, Santa Clara, CA) using MRM to measure

240 endogenous water-soluble metabolites, providing a steady-state analyses of the samples. The 240 compounds were chosen due to their involvement in central pathways important in a number of malignancies. The source parameters were as follows: gas temperature, 250 °C; gas flow rate, 14 L/min; nebulizer, 20 psi; sheath gas temperature, 350 °C; sheath gas flow rate, 12 L/min; capillary, 3000 V positive and 3000 V negative; nozzle voltage, 1500 V positive and 1500 V negative. Approximately 8–11 data points were acquired per detected metabolite. Samples were delivered to the mass spectrometer via normal phase chromatography using either a 4.6 mm ID × 10 cm Amide XBridge HILIC column (Waters) or a Luna 3 µm NH2 100 Å column (Phenomenex) at 300 µL/min (refer to Supplementary Table 2A for detailed LC/MS platforms). Gradients were run from 85% buffer (B; HPLC grade acetonitrile or 0.1% formic acid in acetonitrile) to 35% B from 0–3.5 min, 35% B to 2% B from 3.5–11.5 min, held in 2% B from 11.5–16.5 min, from 2% B to 85% B from 16.5–17.5 min, and held in 85% B for 7 min to re-equilibrate the column. The peak area for each metabolite was integrated using Mass Hunter Workstation Software Quantitative Analysis Version B.06.00 software (Agilent Technologies, Santa Clara, CA).

Metabolic Data Analysis and Associated Statistical Analysis: The detection level of the MS data was set at 100, i.e., expression levels below 100 were considered missing values. Specifically, metabolites with 30% or more missing values in the PCa or adjacent benign tissues were discarded from the analysis. Missing values in the remaining 190 metabolites were imputed using the k-nearest neighbor method (k = 5). The data were log2 transformed and centered using the median level of spiked internal standards on a per-sample, per-method basis. For every metabolite in the normalized

dataset, two-sided paired *t*-tests were conducted to compare its expression level in the PCa and adjacent benign tissues, separately for the different racial groups and for the pooled sample. Since we have compared the metabolite levels between prostate cancer and patient-matched benign adjacent tissue, and since these data were normally distributed, we have used paired t-test to compute the significance. Differential metabolites were identified by adjusting the p-values for multiple testing at an FDR threshold of 30% using the Benjamini-Hochberg procedure. The results were visualized using heat maps implemented in the R package 'gplots'.

Enrichment analyses of the metabolic pathways were conducted using the R package NetGSA developed in [4, 5]. NetGSA is a network topology-based pathway enrichment method that incorporates available a priori information on connections between biomolecules in a mixed-effect linear regression framework and conducts formal hypothesis tests for pathway enrichment. In this pathway enrichment analysis, we used a network of biochemical reactions among metabolites obtained from the Kyoto Encyclopedia of Genes and Genomes (KEGG) and used in [6]. In this network, each node represents a metabolite, and an edge between two metabolites indicates that there are one or more biochemical reactions with these two metabolites appearing as a substrate-product pair.

Importantly, 190 metabolites identified in this study mapped to 197/236 KEGG pathways, giving a coverage of ~83.5%. Notably, the average size of each pathway with KEGG is relatively small. This reduces the possibility of obtaining significant enrichments using a conventional GSEA/GSA type enrichment analysis. NetGSA, in contrast, uses interactions between members of each pathway. It is also based on a

self-contained null hypothesis. The latter has proven to be robust when examining small size pathways [7]. Furthermore, even in presence of large size pathways (sets), competitive methods have been found to exhibit poorer performance compared to self-contained methods [8].

The networks of enriched pathways (FDR ≤ 0.3) were visualized using the Kamada-Kawai layout algorithm implemented in the R package ‘igraph’. Each node represents a metabolic pathway, with node sizes indicating statistical significance ($-\log_{10}$ adjusted p-values larger nodes correspond to lower p-values). Nodes are colored green/red depending on whether they are up/down-regulated in cancer. An edge is drawn between two pathways if they have one or more metabolites in common. The width of an edge indicates the number of common metabolites between two pathways.

Relative measurement of plasma methionine and homocysteine: Relative levels of methionine and homocysteine in this study were measured using an in-house LC-MRM method. Briefly, 30uL of plasma was mixed with 200uL of an equimolar mixture containing two standard compounds ([$15N_2$] Tryptophan and Zeatine) suspended in an equal volume of water and methanol. The aqueous extract was deproteinized using a 3-KDa molecular filter (Amicon Ultracel-3K membrane, Millipore Corporation, Billerica, MA) and the filtrate containing the metabolites was dried under vacuum (Genevac EZ-2plus, Gardiner, NY). Next, 50 µL of 5% phenylisothiocyanate solution containing equal volumes of ethanol, water, pyridine was added to the dried tubes. Samples were incubated for 20 min at room temperature (RT) and then dried under vacuum for 30 min. 40 µL of 5 mM solution of ammonium acetate in methanol was added and the sample was vortexed for 10 min.

20 μL of the derivatized sample was delivered to a ABI-QTRAP 6500 mass spectrometer (Sciex, Concord, Ontario, CA) via reverse phase chromatography using Agilent Zorbax Eclipse XDB C18 (4.6 × 150 mm; 5 μm). The LC mobile phase used 0.2% formic acid in water (A) and 0.2% formic acid in acetonitrile (B) for the analysis. Gradients began with 100% buffer A for 0–5.5 minutes, followed by 100% A to 5% A from 5.5–6.5 minutes, and then held in 100% A from 7–9.5 minutes. The flow rate was 0.5 mL/min. Relative levels of methionine and homocysteine were measured using the MRM transitions 285.0→104 and 271.2→90.1, respectively. Batch effect in the homocysteine data was corrected using Combat method 6.

Relative Quantification of sarcosine in urine samples: A total of 39 AA and 37 EA urine samples were analyzed by GC-MS for sarcosine measurement. Urine supernatants were diluted with methanol containing spiked labeled internal standards (d3-sarcosine and d-creatinine), and extracted overnight at 4°C, using 1:1 molar ratio of water/chloroform at room temperature, with constant shaking. The aqueous methanolic layer was recovered, dried and azeotroped twice by adding 100μL dimethylformamide (DMF), mixed and dried using a speedvac for 30 mins. Following this, 100μL of DMF and N-methyl-N-tert-butylmethylsilyltrifluoroacetamide (MtBSTFA) + 1% t-butyl-dimethylchlorosilane were added and incubated at 60°C for 1 hr. The samples were resuspended in ethyl acetate and injected into a GC-MS. Selective Ion Monitoring (SIM) was used for quantification. Importantly, both these metabolites were baseline separated prior to their quantification. The amount of sarcosine in the sample was calculated by measuring the peak area of the native sarcosine (m/z = 232) to that corresponding to spiked isotope-labeled sarcosine (m/z = 235). Following this, the ratio

of normalized sarcosine to normalized creatinine was calculated.

Tissue Microarray Analysis: Tissue microarrays were obtained from the Pathology and Histology Core at the Baylor College of Medicine. Tissues were de-paraffinized in xylene and rehydrated in a graded alcohol series. The slides were pressure-cooked for 10 min to retrieve the BHMT and CBS antigens. Endogenous peroxidase activity was quenched with 3% hydrogen peroxide for 5 min. Slides were blocked with 3% goat serum at RT for 1 h in a humidity chamber before staining with BHMT (1:50 dilution; mouse monoclonal; Origene Cat# TA500961) and CBS (1:100 dilution; rabbit polyclonal; Abgent, Cat# AP6959) antibodies. The HRP-conjugated goat anti-mouse/anti-rabbit secondary antibodies (Jackson Immunoresearch Laboratories Inc, West Grove, PA) were applied for 40 min. The antigen-antibody reaction was visualized after applying diaminobenzidine for 7 min, and the slides were counterstained with hematoxylin for 1 min. Positive controls were included in each staining run; negative controls were obtained by omitting the primary antibody. Slides were then dehydrated in an alcohol series and cleared in three xylene baths before being mounted with Permount media. Each stain was scored by an experienced genitourinary pathologist in the core lab, who assigned each sample an intensity and extent score. These two values were multiplied (intensity x extent) to produce a final score for each core sample. These final scores were used for the statistical analysis using the Wilcoxon rank sum test. We have included a frequency plot in **Supplementary Figure 5** showing the distribution of the staining scores for CBS and BHMT, in the tissue microarrays, for AA and EA PCa samples.

Summary of Statistical Methods: For normally distributed tissue metabolic data containing matched prostate cancer and benign adjacent tissue, two-sided paired t-test (cancer vs benign) was used. Two-sided Wilcoxon test was used for plasma and urine metabolomics data and tissue microarray datasets. Correlation of West African ancestry with methionine and homocysteine levels was carried out using a non-parametric Spearman rank test. Here both ancestry and metabolite levels were considered as continuous variables. In addition, the ancestry data across all subjects in the case-control cohort was also stratified into tertiles (to ensure that some of the comparisons groups are balanced for the number of self-reported AA) and tested for significance with the Wilcoxon rank sum test. Results showing correlation between West African ancestry and plasma methionine/homocysteine levels are represented using scatter plots.

Analysis examining correlation between dietary variables and plasma methionine/ homocysteine levels was carried out using linear regression based multivariable analysis after controlling for covariates including race, BMI and age at diagnosis. The nutritional variables considered for the analysis were restricted to those for which we had enough number of responses (refer to dietary questionnaire at the end of Supplementary Methods section). In addition, we combined the different ordinal data points to dichotomize each of the specific nutrient variable into a low and a high group as listed below.

**Variable (number of responses in**	**High Group**	**Low Group**

**parenthesis)**		
Amount of Meat Consumed per serving (bacon, pork, lamb, chicken)	≥ 7 ounces. (28)	≤ 6 ounces. (64)
Method used for cooking meat	Fried. (11)	Boil, Bake, Broil, Grill, Steam and Microwave. (63)
Red meat doneness	Well done. (50)	Medium, Rare and never eats meat. (43)
Fish consumption frequency	Daily, more 2-3 times per week and 4-6 times per week. (28)	Once per week, 1-3 per month, never or less than once a month. (65)
Amount of Fish Consumed per serving	>12 ounces, 7-12 ounces. (31)	3-6 ounces, < 3 ounces. (62)
Consumption of fat-butter	Yes (39)	No (39)
Consumption of fat-bacon	Yes (13)	No (32)
Consumption of fat-margarine	Yes (40)	No (36)
Consumption of fat-olive oil	Yes (57)	No (25)
Consumption of fat-Canola oil	Yes (39)	No (38)
Consumption of fat-Other oil	Yes (36)	No (41)

Amount of Vegetables Consumed per serving	2-cups or more, and between 1 and 2 cups. (58)	½ cup to 1 cup, less than1/2 cup and none. (35)
Amount of Broccoli Consumed per serving	More than 1 cup. (14)	Less than 1 cup. (58)
Amount of Garlic Consumed per week	More than one clove.(40)	None. (53)
Amount of Onion Consumed per week	More than one onion. (45)	Less than one onion. (48)
Frequency of Allium per month	More than once a month. (33)	Never or less than once a month. (60)
Amount of fresh tomatoes Consumed per week	> 3 (34)	≤ 2 (58)
Amount of processed tomatoes Consumed per week	More than once per week. (29)	Once per week or 1-3 times per month or never. (63)
Amount of Ketchup	More than once per week. (60)	Once per week or 1-3 times per month or never. (20)
Nutritional status	Good. (60)	Fair. (17)

A multivariable analysis was carried out controlling for race, BMI and age at diagnosis, each time adding one variable into the model and testing it in PCa cases and controls, independently (refer **Supplementary Table 10-12)**. Importantly, only a few dietary variables had enough responses to be tested using this
multivariable linear regression approach. This is a limitation of this study due to which we were unable to accurately assess for dietary associations with methionine and homocysteine levels in this data set and hence included dietary association analysis in the **Supplementary Table 10-12**.

Importantly, in all cases, including enrichment analysis, Benjamini-Hochberg (BH) method was used to compute the false discovery rate (FDR) [9].

Study approval: All human samples were obtained in a de-identified manner with appropriate patient consent and IRB-approval of Baylor College of Medicine.

Nutritional Questionnaire used for the NCI-Maryland Prostate Cancer Case-Control Study

1. During the past 6 months, how often have you eaten meat? (*Includes chicken, beef, pork and lamb but not fish*)

( )0 daily

( )1 4-6 per week

( )2 2-3 per week

( )3 once per week

( )4 1-3 per month

( )5 never or less than once a month

2. Two years ago, how often did you eat meat? (*Includes chicken, beef, pork and lamb but not fish*)

( )0 as frequently as it has been in the past 6 months

( )1 daily

( )2 4-6 per week

( )3 2-3 per week

( )4 once per week

( )5 1-3 per month

( )6 never or less than once a month

3. How much meat do you usually eat per serving?(*Includes chicken, beef, pork and lamb but not fish*)

For help: three ounces of meat is about the size of a cassette tape or a deck of cards.

( )0 more than 12 ounces

( )1 7-12 ounces

( )2 3-6 ounces

( )3 less than 3 ounces, but still eats meat

( )4 never eats meat

4. During the past 6 months, how often have you eaten beef or lamb (includes steaks, stew, hamburger, roast, or hotdog)?

( )0 daily

( )1 4-6 per week

( )2 2-3 per week

( )3 once per week

( )4 1-3 per month

( )5 never or less than once a month

5. During the past 6 months, how often have you eaten pork (includes bacon, chops, roast, or sausage)?

( )0 daily

( )1 4-6 per week

( )2 2-3 per week

( )3 once per week

( )4 1-3 per month

( )5 never or less than once a month

6. During the past 6 months, how often have you eaten poultry (includes chicken, turkey, or duck)?

( )0 daily

( )1 4-6 per week

( )2 2-3 per week

( )3 once per week

( )4 1-3 per month

( )5 never or less than once a month

7. How is your meat usually cooked? (*Includes chicken, beef, pork and lamb but not fish*) (Code all that apply)

( )$_0$ never eats meat (**skip to question 10**)

( )$_1$ eats meat

	How is your meat usually cooked?	
a.	baked	( )$_0$ no ( )$_1$ yes
b.	boiled	( )$_0$ no ( )$_1$ yes
c.	fried	( )$_0$ no ( )$_1$ yes
d.	grilled	( )$_0$ no ( )$_1$ yes
e.	steamed	( )$_0$ no ( )$_1$ yes
f.	microwaved	( )$_0$ no ( )$_1$ yes
e.	broiled	( )$_0$ no ( )$_1$ yes

8. Which method do you use most often? (*Includes chicken, beef, pork and lamb but not fish*)

( )$_0$ baked

( )$_1$ boiled

( )$_2$ fried

( )3 grilled

( )4 steamed

( )5 microwaved

( )6 broiled

( )7 never eats meat

9. The red meat you eat is usually (*Includes beef and pork*)

( )0 well done

( )1 medium

( )2 rare

( )3 never eats meat

10. How often do you eat fish? (*Fresh fish, not canned fish*)

( )0 daily

( )1 4-6 per week

( )2 2-3 per week

( )3 once per week

( )4 1-3 per month

( )$_5$ never or less than once a month

11. How much fish do you usually eat per serving?

For help: three ounces of grilled fish is the size of a typical checkbook.

( )$_0$ more than 12 ounces

( )$_1$ 7-12 ounces

( )$_2$ 3-6 ounces

( )$_3$ less than 3 ounces

12. What kinds of fat is used in the foods you eat? (Code all that apply)

( )$_0$ none (**skip to question 13**)

( )$_1$ eats fat

	What kinds of fat used in the foods you eat?	
a.	butter	( )$_0$ no ( )$_1$ yes
b.	bacon-fat	( )$_0$ no ( )$_1$ yes
c.	margarine	( )$_0$ no ( )$_1$ yes
d.	olive oil	( )$_0$ no ( )$_1$ yes

e.	canola oil	( )$_0$ no ( )$_1$ yes
f.	other oils	( )$_0$ no ( )$_1$ yes

13. During the past 6 months, how often did you have bacon-fat or drippings in your meals (includes breakfast, lunch, dinner)?

( )$_0$ two-times or more per day

( )$_1$ once per day

( )$_2$ 4-6 per week

( )$_3$ 2-3 per week

( )$_4$ once per week

( )$_5$ less than once per week

( )$_6$ none or less than once per month

14. Two years ago, how often did you have bacon-fat or drippings in your meals?

( )$_0$ as frequently as it has been in the past 6 months

( )$_1$ twice per day

( )$_2$ once per day

( )$_3$ 4-6 per week

( )4 2-3 per week

( )5 once per week

( )6 less than once per week

( )7 none or less than once per month

15. During the past 6 months, how much butter have you eaten per week?

For help: eight tablespoons of butter are equal to a stick of butter

( )0 more than 24 tablespoons (or more than 3 sticks)

( )1 17-24 tablespoons (or 2-3 sticks)

( )2 9-16 tablespoons (or 1-2 sticks)

( )3 8 tablespoons or less (or less than a stick)

( )4 none

16. Two years ago, how much butter did you eat per week?

( )0 more than 24 tablespoons (or more than 3 sticks)

( )1 17-24 tablespoons (or 2-3 sticks)

( )2 9-16 tablespoons (or 1-2 sticks)

( )3 8 tablespoons or less (or less than 1 stick)

( )4 none

17. During the past 6 months, how often have you eaten vegetables (includes garlic, onions)?

( )$_0$ daily

( )$_1$ 4-6 per week

( )$_2$ 2-3 per week

( )$_3$ once per week

( )$_4$ 1-3 per month

( )$_5$ never or less than once a month

18. Two years ago, how often did you eat vegetables (includes garlic, onions)?

( )$_0$ as frequently as it has been in the past 6 months

( )$_1$ daily

( )$_2$ 4-6 per week

( )$_3$ 2-3 per week

( )$_4$ once per week

( )$_5$ 1-3 per month

( )$_6$ never or less than once a month

19. How many vegetables do you usually eat per serving?

For help: Your fist is approximately one cup.

( )0 2 cups or more

( )1 between 1 and 2 cups

( )2 ½ cup to 1 cup

( )3 less than ½ a cup

( )4 none

20. How are your vegetables usually cooked?

( )0 steamed

( )1 sauteed

( )2 boiled

( )3 fried

( )4 microwaved

( )5 fresh/uncooked

( )6 never eats vegetables

21. Over the past 6 months, how often did you eat broccoli (fresh or frozen)?

( )0 never **(Skip to question 23)**

( )1 less than once per month

( )2 2-3 times per month

( )3 1 time per week

( )4 2 times per week

( )5 3-4 times per week

( )6 5-6 times per week

( )7 1 time per day

( )8 2 or more times per day

22. Each time you ate broccoli, how much did you usually eat?

For help: Your fist is approximately one cup.

( )0 Less than 1/4 cup

( )1 1/4 to 1 cup

( )2 More than 1 cup

23. During the past 6 months, how often have you eaten garlic?

( )0 daily

( )1 4-6 per week

( )2 2-3 per week

( )3 once per week

( )4 1-3 per month

( )5 never or less than once a month

24. Two years ago, how often did you eat garlic?

( )0 as frequently as it has been in the past 6 months

( )1 daily

( )2 4-6 per week

( )3 2-3 per week

( )4 once per week

( )5 1-3 per month

( )6 never or less than once a month

25. How much fresh garlic do you have in your food per week?

( )0 more than 2 heads

( )1 2 heads

( )2 1 head

( )3 half a head

( )4 a clove

( )5 none

26. During the past 6 months, how often have you eaten onions?

( )0 daily

( )1 4-6 per week

( )2 2-3 per week

( )3 once per week

( )4 1-3 per month

( )5 never or less than once a month

27. Two years ago, how often did you eat onions?

( )0 as frequently as it has been in the past 6 months

( )1 daily

( )2 4-6 per week

( )3 2-3 per week

( )4 once per week

( )5 1-3 per month

( )6 never or less than once a month

28. How many onions do you eat with your food per week?

( )$_0$ more than 4 onions

( )$_1$ 3-4 onions

( )$_2$ 2 onions

( )$_3$ 1 onion

( )$_4$ half an onion or less

( )$_5$ none

29. How often do you eat other types of allium vegetables such as leek, chives or scallions?

( )$_0$ daily

( )$_1$ 4-6 per week

( )$_2$ 2-3 per week

( )$_3$ once per week

( )$_4$ 1-3 per month

( )$_5$ never or less than once a month

30. During the past 6 months, how often have you eaten fresh tomatoes?

( )$_0$ daily

( )1 4-6 per week

( )2 2-3 per week

( )3 once per week

( )4 1-3 per month

( )5 never or less than once a month

31. Two years ago, how often did you eat fresh tomatoes?

( )0 as frequently as it has been in the past 6 months

( )1 daily

( )2 4-6 per week

( )3 2-3 per week

( )4 once per week

( )5 1-3 per month

( )6 never or less than once a month

32. How many fresh tomatoes do you eat per week?

( )0 more than 10

( )1 6-10

( )2 3-5

( )3 1-2

( )4 less than one

33. How often do you eat food with processed tomatoes (puree, sauce)?

Examples are: spaghetti or pizza with tomato sauce.

( )0 daily

( )1 4-6 per week

( )2 2-3 per week

( )3 once per week

( )4 1-3 per month

( )5 never or less than once a month

34. How often do you have ketchup with your food?

( )0 daily

( )1 4-6 per week

( )2 2-3 per week

( )3 once per week

( )4 1-3 per month

( )5 never or less than once a month **(Skip to next section)**

35. How much ketchup do you usually eat per meal?

( )0 more than 6 tablespoons

( )1 4-6 tablespoons

( )2 1-3 tablespoons

( )3 less than 1 tablespoon

**NUTRITION** ( )1 Very good ( )2 Good ( )3 Fair ( )4 Poor

REFERENCES

1. Smith CJ, Dorsey TH, Tang W, *et al.* Aspirin Use Reduces the Risk of Aggressive Prostate Cancer and Disease Recurrence in African-American Men. Cancer Epidemiol Biomarkers Prev 2017;26(6):845-853.

2. Bress A, Han J, Patel SR, *et al.* Association of aldosterone synthase polymorphism (CYP11B2 -344T>C) and genetic ancestry with atrial fibrillation and serum aldosterone in African Americans with heart failure. PLoS One 2013;8(7):e71268.

3. Tian C, Hinds DA, Shigeta R, *et al.* A genomewide single-nucleotide-polymorphism panel with high ancestry information for African American admixture mapping. Am J Hum Genet 2006;79(4):640-9.

4. Shojaie A, Michailidis G. Network enrichment analysis in complex experiments. Stat Appl Genet Mol Biol 2010;9:Article22.

5. Shojaie A, Michailidis G. Analysis of gene sets based on the underlying regulatory network. J Comput Biol 2009;16(3):407-26.

6. Kaushik AK, Shojaie A, Panzitt K, *et al.* Inhibition of the hexosamine biosynthetic pathway promotes castration-resistant prostate cancer. Nat Commun 2016;7:11612.

7. Ma J, Shojaie A, Michailidis G. Network-based pathway enrichment analysis with incomplete network information. Bioinformatics 2016;32(20):3165-3174.

8. Tsai CA, Chen JJ. Multivariate analysis of variance test for gene set analysis. Bioinformatics 2009;25(7):897-903.

9. Benjamini Y, Hochberg Y. Controlling the False Discovery Rate: A Practical and Powerful Approach to Multiple Testing. J.R. Statist, Soc, B 1995;57(1):289-300.
